# Targeting HDAC6 improves anti-CD47 immunotherapy

**DOI:** 10.1186/s13046-024-02982-4

**Published:** 2024-02-27

**Authors:** Maria Gracia-Hernandez, Ashutosh S. Yende, Nithya Gajendran, Zubaydah Alahmadi, Xintang Li, Zuleima Munoz, Karen Tan, Satish Noonepalle, Maho Shibata, Alejandro Villagra

**Affiliations:** 1 Department of Biochemistry and Molecular Medicine, The George Washington University, Washington, DC USA; 2 Department of Anatomy and Cell Biology, The George Washington University, Washington, DC USA; 3 Oncology Department, Georgetown University Medical Center, Washington, DC USA

**Keywords:** Histone deacetylases, CD47, Melanoma, Nexturastat A, Phagocytosis, Macrophages, Immunotherapy

## Abstract

**Background:**

Cancer cells can overexpress CD47, an innate immune checkpoint that prevents phagocytosis upon interaction with signal regulatory protein alpha (SIRPα) expressed in macrophages and other myeloid cells. Several clinical trials have reported that CD47 blockade reduces tumor growth in hematological malignancies. However, CD47 blockade has shown modest results in solid tumors, including melanoma. Our group has demonstrated that histone deacetylase 6 inhibitors (HDAC6is) have immunomodulatory properties, such as controlling macrophage phenotype and inflammatory properties. However, the molecular and cellular mechanisms controlling these processes are not fully understood. In this study, we evaluated the role of HDAC6 in regulating the CD47/SIRPα axis and phagocytosis in macrophages.

**Methods:**

We tested the role of HDAC6is, especially Nexturastat A, in regulating macrophage phenotype and phagocytic function using bone marrow-derived macrophages and macrophage cell lines. The modulation of the CD47/SIRPα axis and phagocytosis by HDAC6is was investigated using murine and human melanoma cell lines and macrophages. Phagocytosis was evaluated via coculture assays of macrophages and melanoma cells by flow cytometry and immunofluorescence. Lastly, to evaluate the antitumor activity of Nexturastat A in combination with anti-CD47 or anti-SIRPα antibodies, we performed in vivo studies using the SM1 and/or B16F10 melanoma mouse models.

**Results:**

We observed that HDAC6is enhanced the phenotype of antitumoral M1 macrophages while decreasing the protumoral M2 phenotype. In addition, HDAC6 inhibition diminished the expression of SIRPα, increased the expression of other pro-phagocytic signals in macrophages, and downregulated CD47 expression in mouse and human melanoma cells. This regulatory role on the CD47/SIRPα axis translated into enhanced antitumoral phagocytic capacity of macrophages treated with Nexturastat A and anti-CD47. We also observed that the systemic administration of HDAC6i enhanced the in vivo antitumor activity of anti-CD47 blockade in melanoma by modulating macrophage and natural killer cells in the tumor microenvironment. However, Nexturastat A did not enhance the antitumor activity of anti-SIRPα despite its modulation of macrophage populations in the SM1 tumor microenvironment.

**Conclusions:**

Our results demonstrate the critical regulatory role of HDAC6 in phagocytosis and innate immunity for the first time, further underscoring the use of these inhibitors to potentiate CD47 immune checkpoint blockade therapeutic strategies.

**Supplementary Information:**

The online version contains supplementary material available at 10.1186/s13046-024-02982-4.

## Introduction

One of the latest advances in immunotherapy entails the development of antibodies that block immune checkpoints (IC) exploited by cancer cells for immune evasion. Blocking antibodies targeting adaptive ICs, such as PD-1 and CTLA-4, have improved clinical outcomes for cancer patients as they potentiate antitumor immunity, specifically T cells [1, 2]. Despite the initial clinical success of IC blockade (ICB) for melanoma, approximately half of the patients do not have long-lasting effects [3–5]. Besides evading adaptive immunity, cancer cells can escape innate immune cells such as macrophages or dendritic cells (DCs) via CD47 [6].

The CD47/SIRPα axis is an innate IC that regulates phagocytosis of cancer cells by macrophages and other myeloid cells and is thus exploited by tumors to escape innate immunity. CD47, also known as “do not eat me” signal, prevents the phagocytosis of cancer cells by interacting with signal regulatory protein alpha (SIRPα) on macrophages or other myeloid cells, preventing the cytoskeletal rearrangement needed for phagocytosis [7, 8]. CD47 suppresses other pro-phagocytic signals such as Fcγ receptors, complement receptors, and calreticulin [9, 10]. Therefore, high SIRPα expression correlates with poor prognosis [11].

CD47/SIRPα blockade is being evaluated as a therapeutic target to enhance innate antitumor responses. CD47 blockade induces antibody-dependent cellular phagocytosis (ADCP) and T cell-mediated destruction of immunogenic tumors [12], while targeting SIRPα potentiates innate and adaptive antitumor immunity [13]. Therefore, intensive research is ongoing to target the CD47/SIRPα axis to enhance antitumor immunity [14–17]. However, other researchers have reported that the blockade of innate ICs is insufficient to induce durable antitumor immune responses. Clinical trials showed that combining anti-CD47 with Azacitidine, an epigenetic drug, showed excellent efficacy with tolerable side effects in acute myeloid leukemia patients [16]. Preclinical studies using melanoma models demonstrated that combining anti-CD47 with anti-PD-L1 or antibodies targeting tumor-specific antigens provides more robust antitumor responses [14, 18].

Our group has previously reported that histone deacetylase 6 inhibitors (HDAC6is) are immunomodulators. Specifically, HDAC6is regulate the expression of PD-L1 [19], enhance the immunogenicity of melanoma cells [20], and increase the antitumor efficacy of anti-PD-1 by modulating the M2 phenotype of macrophages [19, 21, 22]. Others have reported that HDACis such as valproic acid and suberoylanilide hydroxamic acid (SAHA), enhance phagocytosis and decrease CD47 expression [23, 24]. Overall, these studies suggest that combining HDACis with anti-CD47 could modulate the CD47/SIRPα axis, enhance phagocytosis, and decrease tumor growth by enhancing the antitumor immune response.

In this study, we evaluate the role of HDAC6is in modulating the phenotype and phagocytic function of macrophages. We observed that HDAC6 inhibition promotes the M1-like and downregulates the M2-like macrophage phenotype. Furthermore, HDAC6is such as Nexturastat A (NextA) downregulate SIRPα on macrophages and modulate CD47 expression in melanoma cells. Modulating the CD47/SIRPα leads to increased phagocytosis in NextA-treated macrophages, which is enhanced with anti-CD47 antibodies. Lastly, we evaluated the antitumor properties of the combination of NextA and anti-SIRPα and NextA and anti-CD47 in vivo using the SM1 melanoma mouse model. We observed that the combination of NextA and anti-SIRPα did not decrease the tumor growth. However, the combination of NextA and anti-CD47 significantly decreased tumor growth, increased immune cell infiltration in the tumor microenvironment (TME), and modulated innate antitumor immunity, especially macrophage and natural killer (NK) cell populations. Therefore, our studies support the rationale for combining HDACis and anti-CD47 to treat melanoma, as this combination targets both the receptor and the ligand of the CD47/SIRPα axis.

## Results

### HDAC6 inhibitors modulate the phenotype of macrophages

Previous studies from our group and others have demonstrated the immunomodulatory properties of HDAC6i in vitro and in vivo. HDAC6 modulates macrophage infiltration [25], activation [26], and expression of immunosuppressive cytokines like IL-10 through STAT3 interaction [27, 28]. NextA, a highly selective HDAC6i [29], significantly decreased CD206+ M2-like macrophages in breast cancer and melanoma tumor models [21, 22].

Macrophages can be phenotypically classified as M1 or M2; however, this is a simplistic classification as macrophages are highly plastic cells and can display heterogeneity [30]. In this study, we evaluated the role of HDAC6i in modulating the phenotype of macrophages polarized to M1 (polarized with IFNγ and LPS), M1(IFNγ) (polarized with IFNγ only), and M2. Two different M1-like phenotypes were included to represent a more potent inflammatory phenotype (M1) and a weakly activated phenotype M1(IFNγ). For these studies, we used bone marrow-derived macrophages (BMDMs) isolated from C57BL/6 mice and immortalized BMDM cell line BMA3.1A7 (abbreviated A31A7) [31]. Macrophages were unpolarized (M0) or polarized to M1, M1(IFNγ), or M2 in the presence or absence of NextA. We first verified the expression of the pan-macrophage marker CD68 in the A31A7 cell line (Supp. Figure 1 A). We polarized A31A7 macrophages towards the M1-like or M2-like phenotypes in the presence of polarizing cytokines and different concentrations of NextA. We observed a NextA dose-dependent increase in the expression of *Cd80* in M1 polarized macrophages (Supp. Figure 1B) and a dose-dependent decrease in *Arg1* expression in M2 polarized macrophages by qRT-PCR (Supp. Figure 1C). These results showed that 5 µM of NextA was sufficient to induce phenotypic changes in both macrophage phenotypes. Therefore, for these and the following experiments, macrophages were treated with 5 µM of HDAC6i, NextA overnight, in addition to 5 µM of NextA added one hour before the addition of polarizing cytokines.

**Fig. 1:**
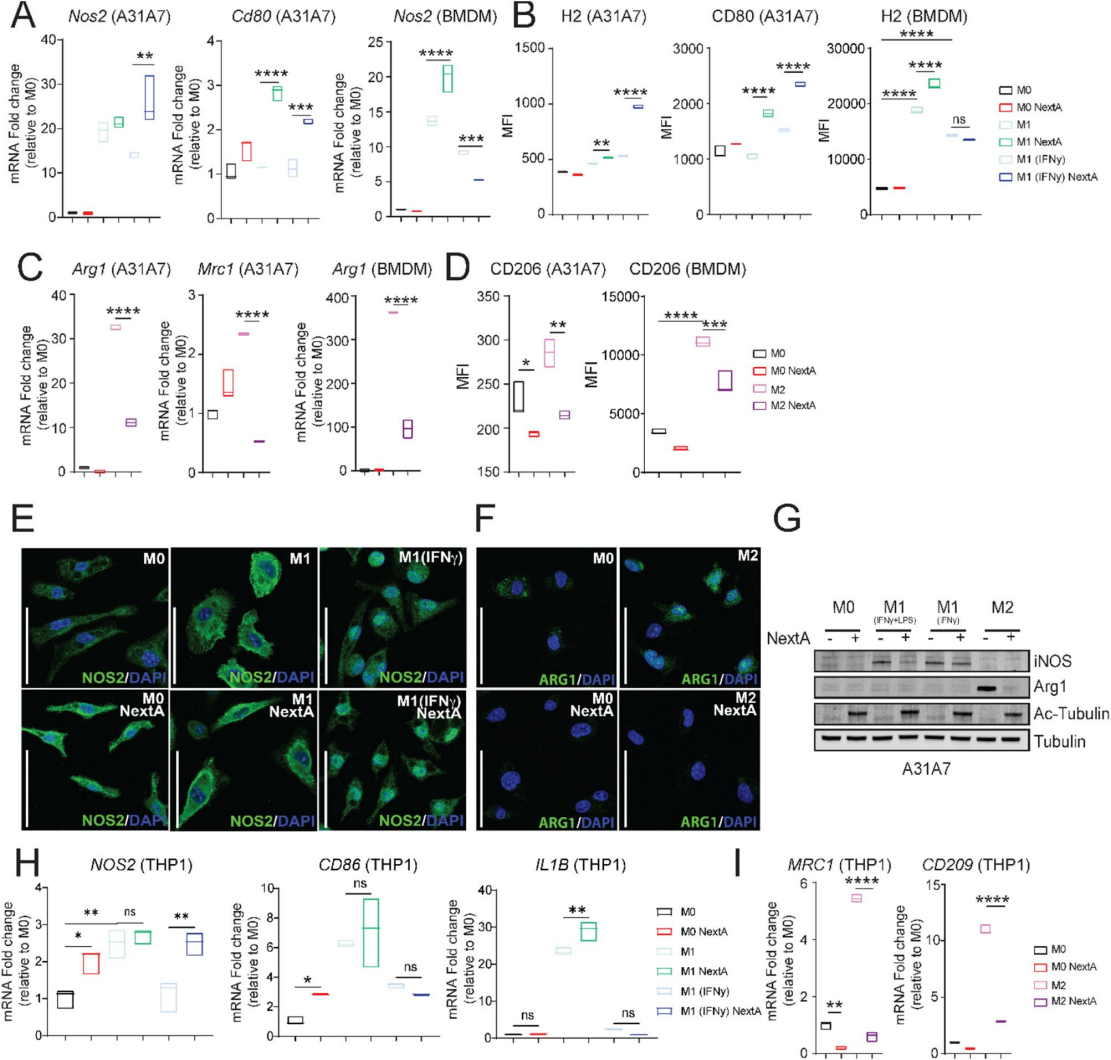
HDAC6 inhibition modulates macrophage phenotype. Macrophages were unpolarized (M0, naïve) or polarized to M1-like phenotypes and M2-like phenotype in the presence or absence of the HDAC6 inhibitor Nexturastat A (NextA, 5 µM). **A** Analysis of M1 phenotype markers *NOS2* and *Cd80* expression of A31A7 macrophages or primary murine BMDMs by qRT-PCR. **B** Analysis of M1 phenotype cell surface markers H2 and CD80 on A31A7 macrophages or murine BMDMs by flow cytometry. **C** Analysis of M2 phenotype marker *Arg1* expression of A31A7 macrophages or BMDMs by qRT-PCR. **D** Analysis of M2 phenotype cell surface marker CD206 on A31A7 macrophages or BMDMs by flow cytometry. **E** Immunofluorescence analysis of M1 marker, iNOS in M1 polarized BMDMs with or without NextA treatment. **F** Immunofluorescence analysis of M2 marker, Arg1 in M2 polarized BMDMs with or without NextA treatment. Nuclei stained with DAPI are shown in blue, iNOS and Arg1 protein staining are shown in green. **G** Western blot analysis of M1 (iNOS) and M2 (Arg1) associated markers. Ac-Tubulin indicated inhibition of HDAC6, and Tubulin is loading control. **H** Gene expression analysis of M1 markers *NOS2*, *CD86*, and *IL1B* in THP-1-derived M1 macrophages and (**I**) M2 markers *MRC1 *(*CD206*) and *CD209* in M2 macrophages by qRT-PCR. Scale bars represent 50 μm. *, *P* < 0.05; **, *P* < 0.01; ***, *P* < 0.001; ****, *P* < 0.0001; ns, non-significant

We then evaluated other M1-associated markers in murine A31A7 macrophages and primary murine BMDMs that were polarized to M1-like. We observed that NextA treatment significantly upregulated *Nos2* and *Cd80* at the transcriptional level (Fig. 1A) and surface expression of Cd80 and H2 by flow cytometry (Fig. 1B). In contrast to the M1 phenotype, NextA significantly downregulated the expression of M2-associated markers such as *Arg1* and *Mrc1 (Cd206)* by qRT-PCR (Fig. 1C) as well as Cd206 surface expression (Fig. 1D) in A31A7 cells and BMDMs by flow cytometry. Furthermore, we evaluated the protein expression of iNOS and Arg1 in BMDMs polarized to M1-like or M2-like phenotypes, respectively (Fig. 1E-F), and observed no changes in iNOS and a substantial decrease in Arg1 by confocal microscopy. We also evaluated these markers by western blotting in A31A7 cells (Fig. 1G) and obtained comparable results with no effect on iNOS protein levels but a significant decrease in Arg1 protein levels with NextA treatment. Hyperacetylation of tubulin indicated inhibition of HDAC6 enzymatic activity.

The effect of HDAC6 enzymatic inhibition over the macrophage phenotype was also validated by testing other HDAC6is Tubacin and Tubastatin A in A31A7 macrophages. Treatment with Tubacin and Tubastatin A upregulated *Cd80* expression in M1-like and downregulated *Arg1* in M2-like macrophages, as evaluated by qRT-PCR (Supp. Figure 1D-E). The selectivity and ability of NextA, Tubacin, and Tubastatin A to downregulate Arg1 expression in M2 macrophages were evaluated by western blot (Supp. Figure 1F). From these results, we determined that NextA is the most potent of HDAC6i assessed, as shown by a robust increase in acetylated tubulin, and decrease in Arg1 expression. Therefore, all subsequent experiments therein were conducted with NextA.

Lastly, we evaluated the modulatory effects of HDAC6i using the human monocytic cell line THP-1. We first verified the differentiation into macrophages by evaluating morphological changes and expression of CD11b, CD14, and CD16 by flow cytometry (Supp. Figure 1G) [32]. Consistent with the mouse macrophage data, we observed that the M1-associated markers *NOS2*, *CD86*, and *IL1B* were either unaffected or significantly upregulated upon NextA treatment (Fig. 1H). NextA significantly downregulated the expression of M2-associated markers such as *MRC1 (CD206)* and *CD209* (Fig. 1I). Tubacin and Tubastatin A downregulated *MRC1* (*CD206*) and *CD209* expression at similar levels as NextA, thus validating our results (Supp. Figure 1H). Altogether, our results indicate that HDAC6 inhibition can be used to modulate the macrophage phenotype.

### Inhibition of HDAC6 decreases SIRPα expression on macrophages

The expression of ICs and immunosuppressive factors can be controlled by master regulators or transcription factors [33]. Previous studies from our lab have demonstrated the role of HDAC6 in modulating the PD-1/PD-L1 pathway [19]. Our group has also reported that combination therapies that target both arms of the PD-1/PD-L1 axis leads to a decrease in tumor growth in melanoma and breast cancer models [21, 22]. Therefore, after assessing the modulatory role of HDAC6 on macrophage phenotype, we evaluated the effects of HDAC6 inhibition in modulating the CD47/SIRPα axis in melanoma cells and macrophages.

**Fig. 2:**
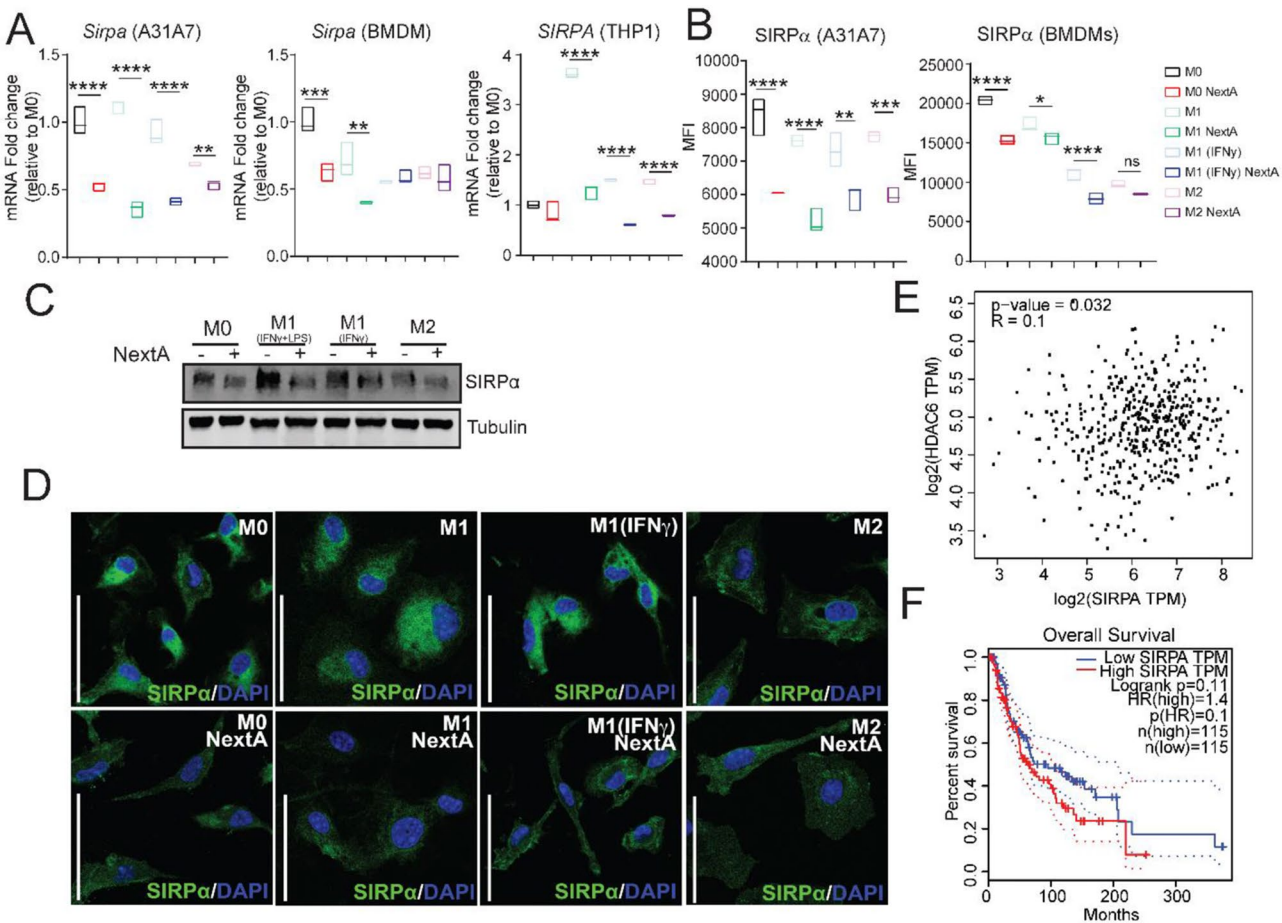
Nexturastat A downregulates SIRPα expression in macrophages. Macrophages were unpolarized (M0, naïve) or polarized to M1-like phenotypes and M2-like phenotype in the presence or absence of Nexturastat A (NextA, 5 µM).**A** Gene expression analysis of SIRPα in A31A7 macrophages (left panel), BMDMs (middle panel), and THP-1-derived macrophages (right panel) evaluated by qRT-PCR. **B** Cell surface expression of SIRPα in A31A7 macrophages (left) and BMDMs (right). **C** Western blots evaluating the SIRPα expression in A31A7 macrophages upon NextA treatment with alpha tubulin as a loading control. **D** Immunofluorescence microscopy representing changes in SIRPα expression (in green) in BMDMs upon NextA treatment. Nuclei were stained with DAPI (in blue). Scale bars represent 50 μm. **E** Pearson correlation between HDAC6 and SIRPα expression in skin cutaneous melanoma patients obtained from the TCGA database through GEPIA. **F** Overall survival of skin cutaneous melanoma patients as it relates to high or low SIRPα expression; data obtained from the TCGA database through GEPIA. *, *P* < 0.05; **, *P* < 0.01; ***, *P* < 0.001; ****, *P* < 0.0001; ns, non-significant

Despite the functional differences among macrophage phenotypes, SIRPα expression is similarly expressed by M1 and M2 macrophages [34]. In this section, we observed that treatment with NextA significantly downregulated *Sirpa* expression on mouse and human macrophages at the transcriptional level (Fig. 2 A). In addition, we observed that treatment with NextA significantly decreased Sirpα expression on the cell surface (Fig. 2B). We further validated our results by assessing Sirpα expression at the protein level by Western Blot in A31A7 macrophages (Fig. 2C), and by confocal microscopy in primary BMDMs (Fig. 2D) and A31A7 cells (Supp. Figure 2A). Overall, *Sirpa* downregulation appears to be due to HDAC6 inhibition and not dependent on the macrophage phenotype, as both unpolarized and polarized macrophages have decreased SIRPα expression upon NextA treatment.

Lastly, we evaluated the correlation between HDAC6 expression and SIRPα expression in skin cutaneous melanoma (SKCM) patients utilizing Gene Expression Profiling Interactive Analysis (GEPIA), a server for interactive analysis of normal and cancer expression profiles [35]. We observed a statistically significant and weak positive correlation between HDAC6 and SIRPα expression in SKCM patients (*p* = 0.032, *R* = 0.1; Fig. 2E), thus supporting our observations. We then evaluated the relationship between low and high SIRPα expression and overall survival of SKCM patients. We observed that, although not statistically significant (*p* = 0.11), SKCM patients with low SIRPα expression tend to have higher survival probability than those with high SIRPα expression (Fig. 2F). These results highlight the importance of the CD47/SIRPα pathway in melanoma and suggest a relationship between HDAC6 and SIRPα expression in SKCM patients, as observed in our results.

To determine if the downregulation of *Sirpa* is exclusive to the HDAC6i NextA, we evaluated *Sirpa* expression upon treatment with other HDAC6 inhibitors, Tubacin and Tubastatin A (abbreviated TubA), by qRT-PCR in A31A7 cells. Treatment with Tubacin and Tubastatin A led to a similar decrease in *Sirpa* expression, suggesting that the observed effects were not an off-target effect of the HDAC6i NextA (Supp. Figure 2B). Furthermore, to investigate whether HDAC6 controls SIRPα expression at the transcriptional level, we cloned the mouse SIRPα promoter into a promoterless pGL4.20[*luc2*/puro] vector to create a SIRPα-Luc reporter plasmid. We transiently transfected A31A7 macrophages in the absence or presence of NextA and observed that NextA significantly decreased SIRPα expression as shown by a decrease in RLU compared to untreated cells (32.2-fold vs. 7.7-fold, Supp. Figure 2C). Overall, these results suggest that HDAC6 may regulate SIRPα expression at the transcriptional level through an unknown mechanism.

We then evaluated the potential role of HDAC6is in regulating the expression of additional pro-phagocytic signals such as *Lrp1, Cd36*, and *Mfge8.* We observed a significant increase in the expression of *Lrp1, Cd36*, and *Mfge8* upon NextA treatment in BMDMs and THP-1-derived macrophages (Supp. Figure 2D-E) and with Tubacin and Tubastatin A (Supp. Figure 2F). Altogether, our results suggest that HDAC6 controls SIRPα expression in macrophages and melanoma patients and that HDAC6i can be used to downregulate the expression of anti-phagocytic signals and upregulate pro-phagocytic signals, which may enhance the phagocytic capacity of macrophages.

### Blocking SIRPα does not affect the antitumor effect of Nexturastat A

Targeting SIRPα through blocking antibodies is being investigated in clinical trials to disrupt the CD47/SIRPα axis [16, 36]. Although anti-SIRPα antibodies induce weak or no phagocytosis, its combination with other agents has proven more successful [36]. Therefore, after observing the potential of HDAC6i to modulate SIRPα expression on macrophages, we evaluated whether HDAC6i would improve SIRPα blockade in vivo.

We inoculated SM1 melanoma cells in C57BL/6 mice (*n* = 11–19 mice per group) and treated with NextA or anti-SIRPα following the experimental outline in Supp. Figure 3A. We observed a significant decrease in tumor growth in the NextA group. However, blocking SIRPα did not result in tumor control in the single-arm group. The mouse anti-SIRPα antibody clone P84 has been reported to have little effect on the CD47/SIRPα interaction, and its ability to decrease tumor growth seems dependent on tumor type and treatment schedule [37, 38]. In addition, combining anti-SIRPα did not enhance the antitumor effect of NextA in the SM1 melanoma model (Supp. Figure 3B-C).

We evaluated the influence of anti-SIRPα and NextA on the immune cell composition (CD45+) of the TME in these tumors. We did not find a significant difference in the number of CD45 + immune cells between the treatment groups (Supp. Figure 3D). However, we observed a significant increase in M1 macrophages in the groups treated with NextA or with the combination of anti-SIRPα and NextA groups compared to anti-SIRPα alone. Regarding M2 macrophages, we only observed a significant decrease with NextA treatment compared to the control group (Supp. Figure 3E-F). This resulted in a significant increase in the M1/M2 ratio of the NextA group, consistent with previous reports (Supp. Figure 3G).

Regarding T cell populations, the combination therapy significantly increased CD4 T cells, with no significant changes in CD8 T cells or T-regs (Supp. Figure 3H-J). Interestingly, the combination therapy increased CD4 and CD8 effector memory T cells but not CD4 and CD8 central memory T cells (Supp. Figure 3K-N). Overall, our results suggest that the combination of NextA and anti-SIRPα does not provide an additive effect to reduce tumor growth of SM1 melanoma, potentially due to the downregulation of SIRPα on macrophages by NextA, which might decrease the efficacy of anti-SIRPα. However, we found that the combination modulates M1 macrophages and effector memory CD4 and CD8 T cells.

### Pharmacological and genetic abrogation of HDAC6 controls CD47 expression in melanoma

**Fig. 3:**
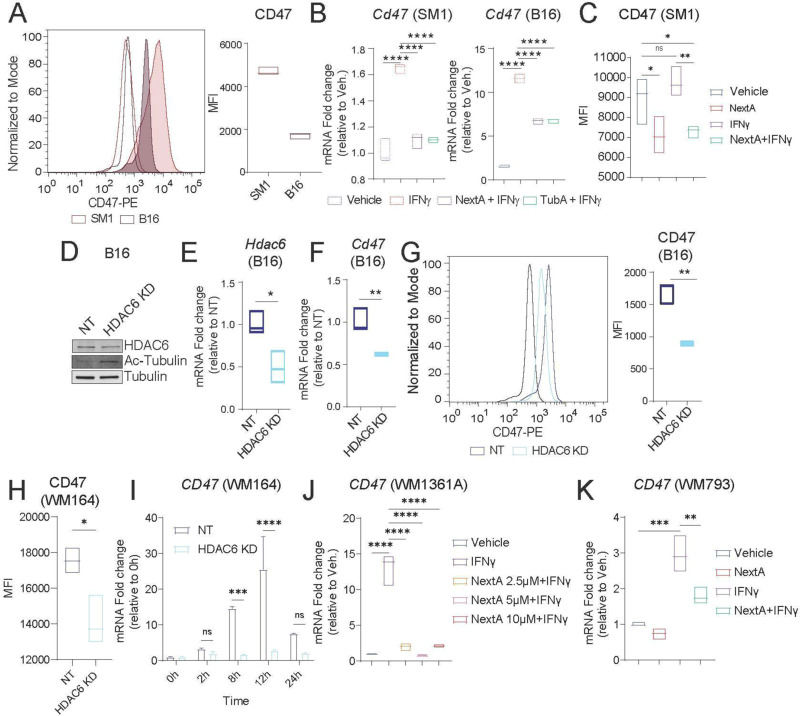
HDAC6 controls CD47 expression in melanoma cells. **A** Comparison of basal expression levels of Cd47 in SM1 and B16 mouse melanoma cells by flow cytometry. **B***Cd47* gene expression analysis of SM1 and B16 cells upon stimulation with IFNγ (100 ng/ml) in the absence or presence of 5 µM of NextA or Tubastatin A (TubA), evaluated by qRT-PCR. **C** Cell surface expression of Cd47 in SM1 cells upon stimulation with IFNγ in the absence or presence of NextA evaluated by flow cytometry. **D** Western Blotting analysis of HDAC6 after performing a partial knockdown (KD) in B16 cells compared to the non-target control (NT). Acetylated tubulin is used as a marker for HDAC6 inhibition, and alpha tubulin is a loading control. **E** qRT-PCR evaluating *Hdac6* expression in B16 NT and HDAC6 KD cells. **F-G** qRT-PCR (**F**) and flow cytometry (**G**) analyses of Cd47 expression in NT and HDAC6 KD B16 cells. **H** Flow cytometry evaluating basal cell surface expression of CD47 in the human melanoma cell line WM164 NT and HDAC6 KD. **I ***CD47* expression analysis of WM164 NT and HDAC6 KD cells upon IFNγ stimulation, evaluated by qRT-PCR at different timepoints. **J** qRT-PCR evaluating *CD47* expression in human melanoma cells WM1361A upon IFNγ stimulation and in the presence of 2.5, 5, and 10 µM of NextA. **K** qRT-PCR evaluating *CD47* expression in WM793 human melanoma cells upon IFNγ stimulation and in the presence of 5 µM of NextA. *, *P* < 0.05; **, *P* < 0.01; ***, *P* < 0.001; ****, *P* < 0.0001; ns, non-significant

We then explored the potential role of HDAC6i in modulating CD47. CD47 is overexpressed in hematological malignancies and solid tumors alike to evade phagocytosis by macrophages [7, 39]. Compared to other cancer types, SKCM ranks within the middle range of CD47 expression [40]. Interestingly, SKCM patients that have low CD47 expression have higher overall survival than those with high expression (*p* = 0.0078, Supp. Figure 3A). Previous publications have demonstrated a limited therapeutic efficacy of CD47 blockade in mice bearing the B16F10 melanoma tumor model [14, 18]. However, the B16F10 melanoma model does not have the mutational burden often seen in human melanoma patients, and it is poorly immunogenic compared to human tumors, thus potentially explaining why B16 models are not responsive to ICB [41]. Better antitumor responses to immunomodulatory agents such as anti-PD-1 have been shown in the SM1 tumor model compared to B16F10 models [21]. Therefore, we evaluated CD47 expression in both mouse melanoma models, SM1 and B16F10 (abbreviated B16). We observed that SM1 cells express CD47 at higher levels than B16F10 cells, as shown in Fig. 3A, suggesting that the SM1 model might be more effective at evaluating the antitumor properties of anti-CD47 than the B16 model.

CD47 can be upregulated by cytokines such as IFNγ, promoting immune evasion [18, 39, 42, 43]. Although the mechanism by which IFNγ drives CD47 upregulation is unclear, IFNγ expression correlates to CD47 expression in SKCM [39]. We first evaluated the kinetics of *Cd47* mRNA in SM1 and B16 cells upon IFNγ stimulation (Supp. Figure 4C-D). Although there is no correlation between HDAC6 and CD47 expression in SKCM patients (*p* = 0.91, *R*=-0.0051, Supp. Figure 4B), we evaluated the role of HDAC6i in abrogating *Cd47* upregulation upon IFNγ stimulation and found that the HDAC6i NextA and Tubastatin A repressed IFNγ-driven *Cd47* expression in SM1 and B16 cells (Fig. 3B-C).

To validate our results, we performed a partial knockdown (KD) of *Hdac6* in B16 cells, as evidenced by a slight decrease in Hdac6 expression and a noticeable increase in acetylated tubulin compared to the non-target (NT) control, as evaluated by Western Blot (Fig. 3D). The partial *Hdac6* KD was also confirmed by qRT-PCR (Fig. 3E). Interestingly, we observed that HDAC6 KD led to downregulation of *Cd47* (Fig. 3F-G, Supp. Figure 4D). Furthermore, we evaluated whether HDAC6 controls CD47 expression at the transcriptional level by generating a CD47-luciferase reporter plasmid. We observed that treatment with NextA repressed IFNγ-mediated upregulation of *Cd47* by measuring luminescence (Supp. Figure 4E). Next, we validated our results using human melanoma cell lines. We observed that HDAC6 KD in WM164 cells downregulated CD47 expression compared to the NT control (Fig. 3H) and prevented IFNγ-driven *CD47* expression at 8 and 12 h post stimulation (Fig. 3I). We further validated our results by treating other human melanoma cell lines, WM1361A and WM793, with IFNγ and NextA (Fig. 3J-K).

Altogether, our results suggest that HDAC6 plays a role in regulating CD47 expression in melanoma cells and that HDAC6i can prevent IFNγ-driven upregulation of CD47.

### Treatment with Nexturastat A increases the phagocytic capacity of macrophages

CD47 blockade enhances phagocytosis by preventing interaction between CD47 and SIRPα, thus allowing the “eat me” signals to dominate [44, 45]. Our results demonstrate that HDAC6i decreased SIRPα expression on macrophages, suggesting that the combination of CD47 blocking antibodies and HDAC6i could enhance the dominance of the pro-phagocytic signals in the phagocytic synapse. Thus, we hypothesized that macrophages treated with NextA would be more phagocytic than untreated macrophages in the presence of anti-CD47.

**Fig. 4:**
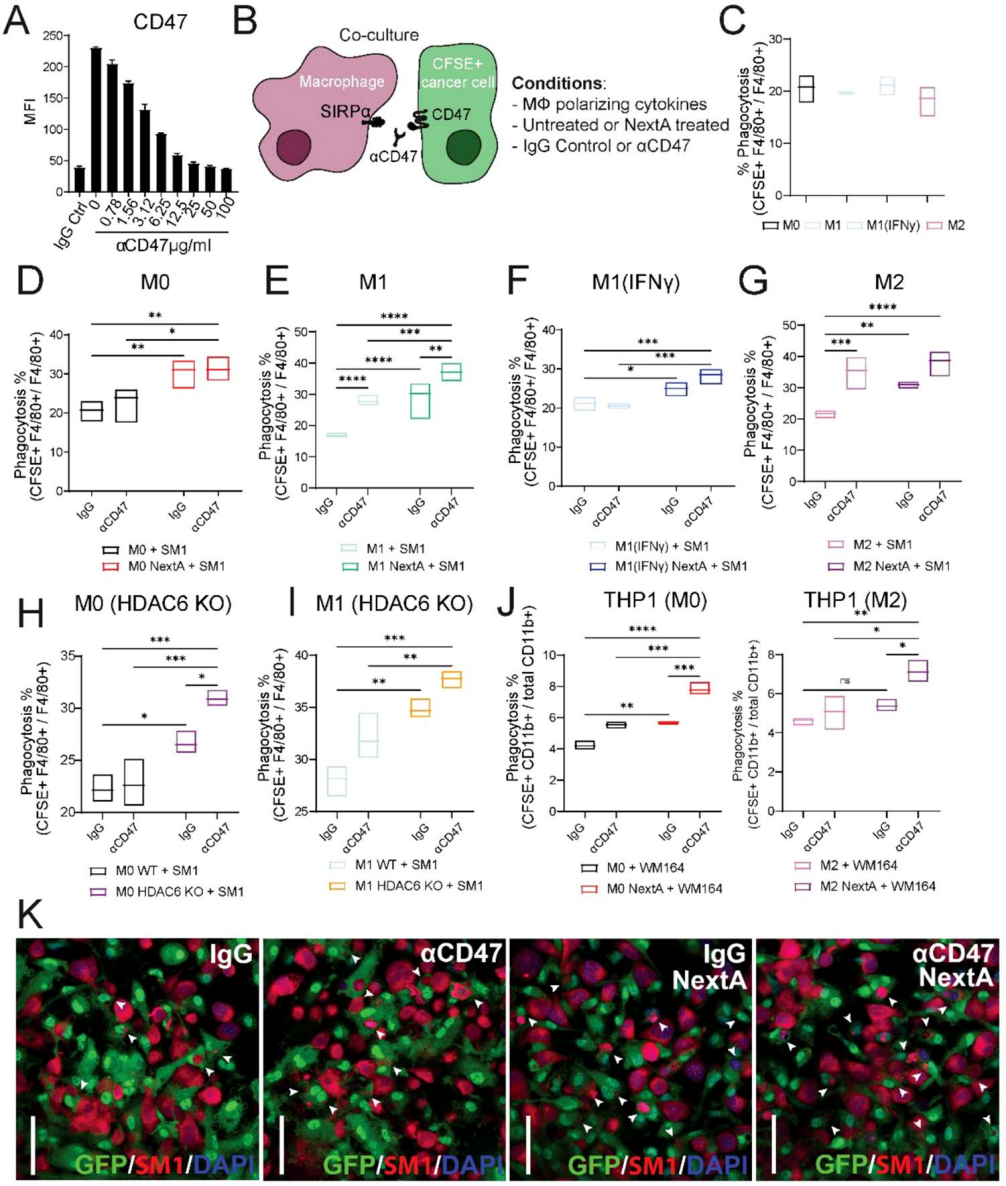
Nexturastat A enhances the phagocytic capacity of macrophages. **A** CD47 antibody titration (miap301) in SM1 cells by flow cytometry. Graph shows unblocked CD47 expression on melanoma cells at different concentrations of the anti-CD47 antibody. **B** Schematic representation of conditions used in phagocytosis assays. For figure panels C through K, BMDMs or THP-1-derived macrophages were unpolarized (M0) or polarized to M1-like or M2-like phenotype in the presence or absence of Nexturastat A (NextA, 5 µM). For figure panels C through J, melanoma cells were stained with CFSE and cocultured at a 2:1 ratio with macrophages, and phagocytosis was analyzed by flow cytometry. **C** Comparison of phagocytosis rates of M0, M1-like or M2-like BMDMs cocultured with CFSE stained SM1 cells. **D-G** Phagocytosis assays of untreated or NextA treated BMDMs cocultured with CFSE stained SM1 cells in the presence or absence of anti-CD47 or IgG isotype control (25 µg/ml). **H-I** Phagocytosis assays of BMDMs harvested from wild type C57BL/6 mice or HDAC6 knockout (KO) mice in the presence of anti-CD47 or isotype control. **J** Phagocytosis assays of THP-1-derived macrophages cocultured with WM164 human melanoma cells in the presence of human anti-CD47 or isotype control. All flow cytometry-based phagocytosis assays are quantified as % CFSE+ F4/80+ or CFSE+ CD11b+ cells out of total F4/80+ or CD11b+ cells. **K** Representative images of phagocytosis assays performed by confocal microscopy using BMDMs isolated from GFP mice (in green) and SM1 melanoma cells stained with CellTrace Far Red (shown in red). Nuclei were stained with DAPI (shown in blue). White arrow heads represent internalization of SM1 cells by macrophages. Scale bars represent 50 μm. *, *P* < 0.05; **, *P* < 0.01; ***, *P* < 0.001; ****, *P* < 0.0001; ns, non-significant

We first determined the concentration of anti-CD47 that allows for the greatest CD47 blockade and epitope saturation in the SM1 cells in vitro, using a previously described method [46] (Fig. 4A, Supp. Figure 5A). After determining the optimal concentration (25 µg/ml), we performed flow cytometry-based phagocytosis assays by staining SM1 cells with CFSE prior to coculture with macrophages in the presence or absence of NextA and/or anti-CD47 or isotype (IgG) control (Fig. 4B). After incubation, cocultured cells were stained for F4/80 and phagocytosis was determined by calculating the percentage of CFSE+ F4/80+ out of total F4/80+ macrophages (Supp. Figure 5B). We first determined the optimal ratio of cancer cells to macrophages using A31A7 macrophages, which was 2:1 across different phenotypes, as there were no significant differences between 3:1 and 2:1 ratio (Supp. Figure 5C-E). We then compared the phagocytic capacity of primary BMDMs left unpolarized (M0) or polarized to M1, M1(IFNγ), or M2, and found similar phagocytosis rates (Fig. 4C), consistent with previous studies demonstrating that both M1 and M2 macrophages are phagocytic [47].

We then evaluated how NextA could modulate the phagocytosis in the presence of anti-CD47 or IgG control. Interestingly, we observed that anti-CD47 did not increase phagocytosis by M0 and M1(IFNγ), whereas it increased phagocytosis by M1 and M2 macrophages (Fig. 4D-G), consistent with previous research [48]. However, treatment with NextA and/or anti-CD47 significantly increased phagocytosis of all macrophage phenotypes. Additionally, we observed comparable results using A31A7 macrophages (Supp. Figure 5F). In addition, our results indicate that NextA treatment of SM1 cells did not have any effect on phagocytosis in M1 macrophages (Supp. Figure 5G), thus suggesting that the increase in phagocytosis could be driven by SIRPα downregulation. We assessed our hypothesis using BMDMs isolated from HDAC6 knockout (KO) mice to validate our results. We observed that HDAC6 KO M0 and M1-like macrophages have a higher phagocytic capacity than their wild-type counterparts and that phagocytosis was further increased with anti-CD47 in the HDAC6 KO macrophages (Fig. 4H-I). Furthermore, we validated our results using THP-1-derived macrophages and WM164 cells. We observed similar results, where NextA treatment significantly increased phagocytosis, an effect that was further enhanced with CD47 blocking antibodies (Fig. 4J).

To further characterize this phenomenon, we evaluated phagocytosis using confocal microscopy (Fig. 4K). For these experiments, BMDMs were harvested from UBC-GFP mice and polarized to the M1 phenotype in the presence of vehicle or NextA and IgG control or anti-CD47. SM1 cells were stained with CellTrace Far Red and cocultured with GFP expressing M1-like macrophages for 2 h at a 2:1 ratio. Phagocytosis was determined by the internalization of the CellTrace Far Red dye in the GFP macrophages and marked with white arrow heads (unmarked figures included in Supp. Figure 5I). As shown in Fig. 4K, M1 NextA + anti-CD47 had the highest number of phagocytic events compared to either treatment alone, thus validating previous results. Using a similar approach, we assessed the kinetics of phagocytosis of untreated and NextA-treated macrophages by performing live cell imaging (Supp. Figure 5H). We observed that anti-CD47 increased phagocytosis compared to the IgG control up to the 180 min timepoint, where both groups reached similar rates. In contrast, NextA-treated M1 macrophages had a slightly higher phagocytosis ratio at the starting timepoint with anti-CD47. However, M1 NextA treated macrophages had the highest phagocytosis rates, which were sustained during the duration of the experiment, irrespective of the antibody. In addition, we tested the effects of blocking SIRPα on the phagocytosis of SM1 melanoma cells by M1 macrophages (Suppl. Figure 6). Similar to Fig. 4K, we observed M1 NextA treated macrophages exhibited the highest phagocytosis when CD47 was blocked, whereas blocking SIRPα on macrophages did not enhance phagocytosis to the extent we observed with M1 NextA and anti-CD47, thus indicating that blocking CD47 on cancer cells is more effective than blocking SIRPα on macrophages to modulate phagocytosis of melanoma cells by macrophages.

In summary, our results suggest that HDAC6i enhances phagocytosis of cancer cells by macrophages, which is further increased upon CD47 blockade. Therefore, this suggests that targeting both sides of the CD47/SIRPα axis could synergistically control tumor growth by enhancing macrophage-mediated antitumor immunity.

### Nexturastat A improves the antitumor effect of anti-CD47 in mouse melanoma models

CD47 blockade aims to enhance phagocytosis by tumor-associated macrophages (TAMs), which display an M2-like phenotype [49]. It is likely that expression of other “do not eat me” signals and/or receptors in the TME, in addition to other immunosuppressive factors, prevents CD47 blockade-mediated phagocytosis by TAMs to later elicit an antitumor adaptive immune response [49]. This supports that anti-CD47 alone is insufficient to decrease tumor burden in melanoma-bearing mice and generate long-lasting antitumor immunity [14, 16, 18]. However, this limitation can be overcome by combining it with therapies that stimulate antitumor immunity, such as antibodies targeting tumor-specific antigens or ICB [14, 18].

HDACi such as SAHA enhance trastuzumab-mediated phagocytosis and thus are strong candidates to use in combination with therapies aiming to enhance phagocytosis [23]. However, to date, there are no studies evaluating the effects of class-selective epigenetic modifiers such as HDAC6 (class IIb) inhibitors in potentiating these effects. Based on our results, we hypothesized that combining HDAC6i with anti-CD47 could result in tumor regression and modulation of the macrophage phenotype in vivo.

**Fig. 5:**
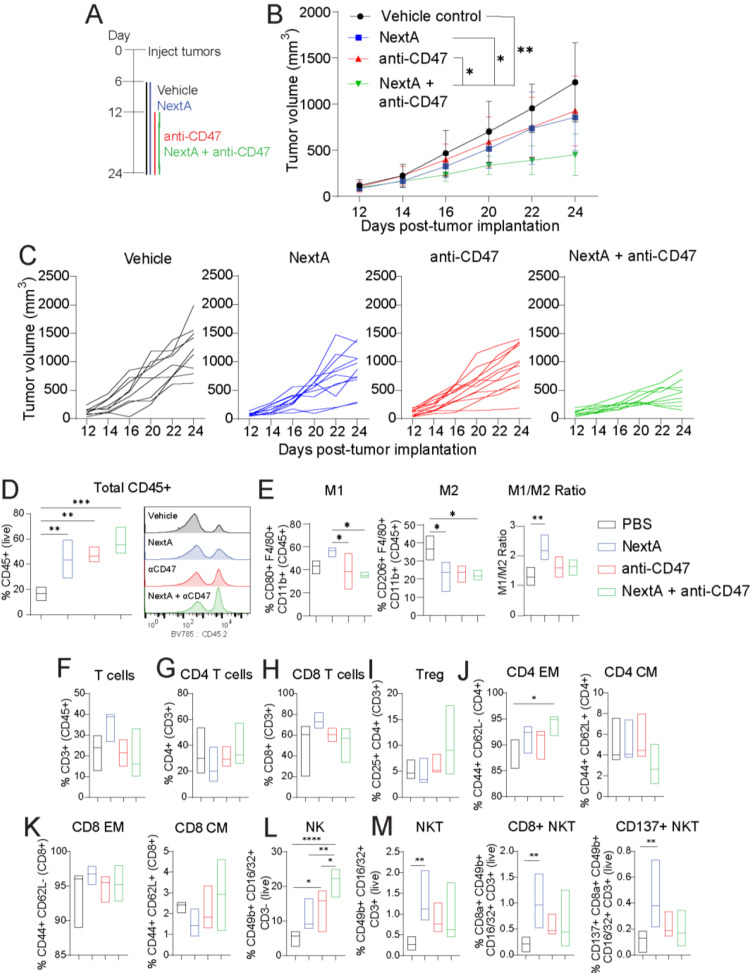
Nexturastat A enhances the antitumor efficacy of anti-CD47 in SM1 melanoma-bearing mice. **A** Schematic representation of treatment timeline. **B** Tumor growth kinetics of SM1 melanoma tumors (*n* = 9–12 mice per group) treated with vehicle control, Nexturastat A (NextA, 20 mg/kg, IP), anti-CD47 (100 µg intratumoral), or combination. **C** Individual tumor growth kinetics in the different groups. Immunophenotyping of tumors was performed by flow cytometry at day 24 post-tumor implantation. The immune cell populations include total CD45+ immune cells (**D**) M1-like, M2-like, and M1/M2 ratio (**E**) total CD3+ T cells (**F**) total CD4+ T cells (**G**) total CD8+ T cells (**H**) T-regs (**I**) effector memory (EM) and central memory (CM) CD4+ T cells (**J**) effector memory (EM) and central memory (CM) CD8+ T cells (**K**) total NK cells NK cells (**L**) and total NKT, CD8+ NKT, and CD137+ CD8 + NKT (**M**). The surface markers used to identify the populations are indicated on the y axis of every graph. *, *P* < 0.05; **, *P* < 0.01; ***, *P* < 0.001; ****, *P* < 0.0001; ns, non-significant

To evaluate our hypothesis, we inoculated C57BL/6 mice with SM1 melanoma cells (*n* = 9–12) and followed the experimental outline in Fig. 5A. The anti-CD47 antibody used in this study is clone miap301, which has been shown to partially exhibit tumor inhibition in syngeneic C57BL/6 mouse models [12]. Anti-CD47 was administered intratumorally to enhance its efficacy and safety, as CD47 is ubiquitously expressed and normal tissues act as an antigen sink [50]. Our results indicate that NextA and anti-CD47 moderately decrease tumor growth as stand-alone therapies compared to vehicle control and that combining these agents further reduces tumor growth in SM1-bearing mice (Fig. 5B-C).

We then evaluated the immunomodulatory effects and immune composition of the TME. Single treatment arms significantly increased total immune cell (CD45+) infiltration, which was further increased in the combination therapy (Fig. 5D). Although not statistically significant, NextA treatment increased M1 macrophages compared to the control (Fig. 5E). Despite the significant decreases in M1 macrophages in the anti-CD47 and combination groups compared to NextA, anti-CD47 did not significantly decrease M1 macrophages when compared to the control. Consistent with previous publications from our group [21, 22], NextA significantly decreased M2 macrophages. CD47 blockade notoriously decreased M2 macrophages (*p* = 0.0583), a statistically significant decrease in the combination group. However, only the NextA group significantly increased the M1/M2 ratio. Altogether, these results support our hypothesis that NextA and anti-CD47 can act in combination in vivo to decrease tumor growth and to modulate the phenotype of TAMs.

We then evaluated the T cell populations in these tumors. We observed that total CD3, CD4, and CD8 T cells and T-regs were not significantly affected in any treatment group (Fig. 5F-I). However, the combination therapy significantly increased CD4 effector memory T cells (Fig. 5J). However, no significant changes were observed in CD4 central memory T cells or CD8 central and effector memory T cells (Fig. 5K).

Lastly, we observed a significant increase in the infiltration of total NK cells with anti-CD47, consistent with findings from other groups [51]. Total NK cell infiltration was increased in the combination group compared to every other group (Fig. 5L). Evaluation of NKT cells revealed that NextA significantly increased total NKT, CD8+ NKT, and CD137+ CD8+ NKT cells in the TME (Fig. 5M).

The antitumor effects of the combination therapy were also observed in the B16F10 melanoma model following the same experimental protocol. Only the combination of NextA and anti-CD47 led to a significant decrease in tumor growth. Regarding the immunophenotyping of tumors, we found no significant differences in the macrophage, T cell, or NK cell populations in the B16 TME by flow cytometry (Supp. Figure 7A-E). The discrepancies between the SM1 and B16F10 models could be explained by the scarce infiltration of immune cells in the TME of B16F10 compared to SM1 (5% vs. 15%, respectively, Supp. Figure 7F), as well as the poor immunogenicity of B16 tumors [41].

Overall, our results show that NextA improves the antitumor efficacy of anti-CD47 in mouse melanoma models. The combination therapy alters the TME, particularly innate immune cells such as macrophages and NK cells. The lack of differences in T cells among groups suggests that the therapies, whether individually or in combination, do not negatively affect T cell infiltration or populations in the TME. However, the antitumor effects observed in these studies could be explained by a modulation of the innate arm of the immune system and suggests that including T cell-targeting strategies could lead to more potent and long-lasting antitumor responses.

## Discussion

Despite the therapeutic success of CD47 blocking antibodies in hematological malignancies, their efficacy is limited in solid tumors. Blocking CD47 or SIRPα cannot induce phagocytosis significantly [36, 52], but combination therapies may help improve these effects. Specifically, combining CD47 or SIRPα blocking antibodies with opsonizing agents is being investigated to augment ADCP and enhance their antitumor effect [13, 37, 53–55]. In melanoma, anti-CD47 alone does not induce lasting antitumor immune responses in B16 tumors unless combined with anti-PD-L1, but not anti-CTLA4 [14, 18]. Other emerging approaches aiming to enhance phagocytosis include using epigenetic modifiers, such as HDACi. Valproic acid and SAHA increase phagocytosis by increasing FcγRIIA expression on monocytes and decreasing CD47 expression [23, 24]. Overall, these studies suggest that combining HDACis with anti-CD47 could augment the therapeutic efficacy of anti-CD47 by modulating phagocytosis and the antitumor response. However, little is known about the role of highly specific HDACis in controlling macrophage phenotype and phagocytosis. Isoform-specific HDACis, such as HDAC6is, have reduced cytotoxicity compared to pan-HDACis [21, 22] and immunomodulatory properties. Our group has shown that HDAC6is regulate PD-L1 in melanoma through STAT3 [19], enhance the immunogenicity of melanoma cells [20], and increase the antitumor efficacy of anti-PD-1 by suppressing M2 macrophages [19, 21, 22].

Taking these observations into account, in the present study, we evaluated whether HDAC6is can modulate the phenotype and phagocytic function of macrophages. We observed that treatment with NextA increased the M1 phenotype and decreased the M2 phenotype of macrophages. More importantly, NextA treatment tilts the balance to a pro-phagocytic milieu by reducing the expression of SIRPα and inducing the expression of other pro-phagocytic signals on macrophages (Fig. 2; Supp. Figure 2). Based on our results, we observed that NextA downregulates SIRPα expression on macrophages independently of their polarization state, whether they may be activated/pro-inflammatory (M1-like) or anti-inflammatory (M2-like). In addition, we found that HDAC6i repressed IFNγ-mediated upregulation of CD47 in mouse and human melanoma cell lines through an unknown mechanism (Fig. 3). However, our luciferase reporter studies suggest that HDAC6 may regulate CD47 expression at the transcriptional level. Although the mechanism by which IFNγ upregulates CD47 is not fully understood, some pathways that are modulated by HDAC6 may be involved in this signaling pathway [39, 43, 56]. For example, our group has shown that HDAC6 interacts with STAT3 and that HDAC6is prevent PD-L1 upregulation upon IL-6 or IFNγ treatment [19]. However, the molecular mechanisms by which HDAC6 regulates the CD47/SIRPα axis remain to be elucidated.

The interaction between CD47 and SIRPα was thoroughly explored by Morrissey et al., where ligation of CD47 with SIRPα at the immune synapse leads to the activation of inhibitory signals that limits macrophage spreading and phagocytosis of the tumor cells [44]. Therefore, downregulation of the CD47/SIRPα axis renders macrophages more phagocytic as less interaction between CD47 and SIRPα can occur, and therefore, less SIRPα-mediated inhibitory cascade occurs. Our phagocytosis assays show that NextA treatment increases phagocytosis of tumor cells by macrophages, which was further enhanced with anti-CD47 (Fig. 4). From our results, one would hypothesize that the increase in phagocytosis in NextA-treated macrophages may be due to the downregulation of SIRPα induced by HDAC6 inhibition. However, previous research has shown that *Hdac6* ^*−/−*^ macrophages have defects in the intracellular killing of phagocytosed bacteria [57] and that loss of HDAC6 impairs the ability of macrophages to phagocytose bacteria [25]. However, it is important to consider that phagocytosis of bacteria is independent of CD47/SIRPα interaction and could be negatively affected by HDAC6 inhibition. As shown in this study, HDAC6 inhibition enhances phagocytosis of SM1 melanoma cells, an event regulated by the CD47/SIRPα axis.

From our results, we hypothesized that the combination of NextA and targeting the CD47/SIRPα axis would decrease SM1 tumor growth. Interestingly, we found that the combination of NextA and anti-CD47 decreased tumor growth in SM1 melanoma-bearing mice, increased total immune cell infiltration in the TME, and altered the immune cell composition compared to the single treatment arms, mostly modulating macrophages and NK cells (Fig. 5). We then assessed if NextA could improve the antitumor effect of anti-SIRPα using the anti-SIRPα antibody clone P84, which has been shown to slow tumor growth of different tumor models [37, 58]. However, we found that NextA does not improve the antitumor efficacy of anti-SIRPα, although the combination had some immunomodulatory properties, such as significantly increasing M1 macrophages as well as effector memory CD4 and CD8 T cells in the TME (Supp. Figure 3). Our hypothesis for the antitumor effects of the combination therapy with NextA and anti-CD47 is that targeting both sides of this innate immune checkpoint has a beneficial effect for antitumor efficacy: downregulation of SIRPα on macrophages through HDAC6 inhibition and blockade of CD47 on tumor cells through the use of blocking antibodies. In addition, the anti-CD47 antibody blocks CD47 on tumor cells, thus coating them with antibodies that can potentially enhance the antitumor immune response. On the contrary, targeting SIRPα through HDAC6 inhibition and anti-SIRPα antibodies only targets one side of the axis, potentially explaining the different results obtained in the respective animal studies. Furthermore, the combination of anti-SIRPα and NextA may not be advantageous because both agents target SIRPα, and NextA might decrease the efficacy of anti-SIRPα antibodies by downregulating its expression.

The combination of NextA and anti-CD47 mostly modulates innate antitumor immunity, as demonstrated by the modulation of macrophages and a significant increase in NK cells. Although SIRPα is an innate IC that has traditionally been associated with myeloid cells, recent studies have demonstrated that primary NK cells express SIRPα, which is detrimental to their cell-killing function, and thus its blockade could augment their antitumor responses [59]. Using a selective HDAC6i, we have observed that NextA downregulates SIRPα expression on NK cells (data not shown). Furthermore, NK cell recruitment, proliferation, and activation are regulated by CD47 [51]. Consistent with other groups, our results show an increase in NK cell infiltration in the TME upon treatment with anti-CD47. Moreover, we observed that the combination of NextA and anti-CD47 increases the infiltration of NK cells in an additive manner. Although our data led us to hypothesize that the combination of NextA and anti-CD47 may modulate NK cells, this study does not provide a full picture of the activation status of NK cells infiltrated in the TME following treatment. Further studies need to be conducted to fully elucidate if the combination of NextA and anti-CD47 can induce the activation of NK cells in vivo and to evaluate whether there is a crosstalk between NK cells and activated macrophages in the combination therapy. Additionally, we observed no significant changes in T-cell populations. It is unclear whether the decrease in tumor growth observed upon CD47 blockade in melanoma models is dependent or independent of T cells (49, 60–61).

In summary, our study represents a novel approach to target the CD47/SIRPα axis by controlling the expression of “do not eat me” signals on melanoma cells and macrophages. To our knowledge, no studies have investigated the potential of HDAC6i in modulating the expression of the CD47/SIRPα pathway. Therefore, the novelty of the present study relies on the use of HDAC6i to control the CD47/SIRPα axis by modulating their expression in melanoma cells and macrophages, respectively. Furthermore, our results provide a rationale for combining CD47 blocking antibodies with HDAC6i to modulate macrophage phenotype, enhance phagocytosis, and potentiate antitumor NK cell responses in vivo.

## Methods

### Cell culture

The bone marrow-derived macrophage cell line BMA3.1A7 was kindly provided by Dr. Kenneth L. Rock (University of Massachusetts Medical School, Worcester, MA) [31] and referred to as A31A7 throughout the text. A31A7 cells were cultured in RPMI 1640 media supplemented with 10% fetal bovine serum (FBS), 1% Penicillin-Streptomycin (30-002-CI, Corning), 1% HEPES buffer (H3537, Sigma Aldrich), 1% L-glutamine (25,030,081, Corning) and 1% non-essential amino acids (NEAA, 25-025-Cl, Corning) at 37 °C and 5% CO_2_. THP-1 monocytes were obtained from ATCC (TIB-202) and cultured in complete RPMI 1640 medium containing 10% FBS, 1% NEAA, and 1% penicillin-streptomycin. SM1 cells were obtained from Dr. Antoni Ribas at the University of California, Los Angeles. B16 and WM164 melanoma cells were obtained from ATCC. SM1 and B16 mouse melanoma cell lines, as well as WM164 human melanoma cells, were cultured in complete RPMI 1640 medium supplemented with 10% FBS, 1% NEAA, and 1% penicillin-streptomycin.

### Macrophage isolation and polarization

BMDMs were isolated from the femurs and tibias of wild-type C57BL/6, HDAC6 knockout (C57BL/6J-Hdac6em2Lutzy/J, 029318, Jackson Laboratory), or UBC-GFP (C57BL/6-Tg(UBC-GFP)30Scha/J, 004353, Jackson Laboratory). Isolated bone marrow cells were cultured in RPMI 1640 complete medium with 10% FBS, 1% NEAA, and 1% penicillin-streptomycin in 5% CO_2_ at 37 °C. The medium was supplemented with murine recombinant M-CSF (20 ng/mL, 576404, BioLegend) to differentiate monocytes into macrophages. Three days after bone marrow isolation, undifferentiated and floating cells were washed with PBS and replaced with fresh RPMI media. A31A7 cells and BMDMs were pretreated with 5 µM of NextA or vehicle overnight, in addition to 5 µM of NextA or vehicle added an hour before the polarizing cytokines. Macrophages were polarized to M1-like phenotype using 50 ng/ml of interferon-gamma (IFNγ, 575,306, BioLegend) and 100 ng/ml of bacterial lipopolysaccharide (LPS, L2880, Sigma Aldrich, St. Louis, MO) for 24 h. To polarize macrophages towards the M2-like phenotype, they were stimulated with 20 ng/ml of IL-4 (214-14, PeproTech) and 20 ng/ml of IL-13 (575904, BioLegend).

THP-1 monocytes were treated with 5 ng/ml of phorbol 12-myristate 13-acetate (PMA) to induce differentiation into macrophages. Once converted to macrophages, media was replaced to remove PMA prior to treatment and/or polarization. Polarization towards M1 and M2 phenotypes was performed with respective recombinant human cytokines from PeproTech: 50 ng/ml of IFNγ (300-02), 20 ng/ml IL-4 (200-04), and 20 ng/ml IL-13 (200-13). 100 ng/ml LPS was also used to polarize toward the M1 phenotype.

### In vivo studies

Animal experiments were performed in accordance with protocol A354 approved by the Institutional Care and Use Committee (IACUC) at The George Washington University, and with protocol 2022-016 at Georgetown University. C57BL/6 4–6 week-old female mice were purchased from Charles River Laboratories (Wilmington, Massachusetts, USA). Mice were injected subcutaneously in their right flank with 0.75 × 10^6^ or 10^6^ in vivo passaged SM1 melanoma cells suspended in 100 µL of PBS (Corning, 21-040-CV) at day 0. Cages were randomly assigned to different treatment groups, and mice were treated intraperitoneally with 100 µL of vehicle control or NextA at 20 mg/kg every other day when tumors became palpable, which was a week after tumor implantation. Six days after the first NextA treatment, mice were also treated intratumorally with 100 µg of αSIRPα (clone P84, BE0322, BioXCell, Lebanon, NH, USA), isotype control (clone HRPN, BE0088, BioXCell), or αCD47 (clone miap301, BE0270, BioXCell) administered in 30 µL every three days until the end of the study. Tumor measurements were taken every other day using caliper measurements and calculated using the formula L × W^2^/2. All animals were routinely monitored for early signs of toxicity. Emphasis was given to mortality, body weight, and food consumption. At the endpoint, a post-mortem evaluation was performed, including a gross visual examination of organs.

**Phagocytosis assays** To evaluate phagocytosis by flow cytometry, SM1 melanoma cells were cocultured with A31A7 cells or BMDMs derived from wild-type C57BL/6 mice at a 2:1 ratio for 2 or 4 h in serum-free media. Twenty four hours prior to coculture, macrophages were polarized to their respective phenotypes as previously described in the presence or absence of NextA, and melanoma cells were untreated or treated with NextA. On the day of the coculture, melanoma cells were stained with 1 µM of CellTrace™ CFSE Cell Proliferation Kit (ThermoFisher, C34554) and were washed extensively to remove excess dye. Phagocytosis assays were carried out in the presence of anti-mouse CD47 (clone miap301, 127502, BioLegend, San Diego, CA) and its respective isotype control (IgG, 400502, BioLegend) or anti-human CD47 (clone CC2C6, 323102, BioLegend) or isotype control (MOPC-21, BE0083, BioLegend) at 25 µg/ml. Coculture was set up in non-cell culture treated 96 U-well plates. After coculture, cells were collected and stained with Brilliant Violet 785 anti-mouse F4/80 (clone BM8, BioLegend) or Brilliant Violet 785 anti-human CD11b (clone ICRF44, 301346, BioLegend). Flow cytometry data was acquired with a BD FACS Celesta Cell Analyzer, and data analysis was performed with FlowJo software version 10.3. Phagocytosis was determined as the percentage of CFSE+ F4/80+ or CFSE+ CD11b+ cells out of total F4/80+ or total CD11b+ cells.

Phagocytosis was also evaluated by confocal microscopy and live imaging. BMDMs derived from UBC-GFP mice were plated in Nunc Lab-Tek™ 8-well chamber slides (177402PK, ThermoFisher). The same treatment and polarization protocols as previously described were followed for these experiments. The day of the coculture, SM1 melanoma cells were stained using CellTrace™ Far Red Cell Proliferation Kit (C34564, ThermoFisher), following manufacturer’s protocol. Cells were extensively washed to remove any excess dye. SM1 cells were cocultured with BMDMs at a 2:1 ratio, respectively, for 1 h in the presence of IgG control or αCD47 (BioLegend). After coculture, slides were fixed in 4% paraformaldehyde. Slides were incubated overnight with primary anti-GFP antibody (Cell Signaling, 2956) at 4 °C. Slides were incubated with goat anti-rabbit IgG conjugated to Alexa Fluor 488 (A11008, ThermoFisher). Slides were mounted using VECTASHIELD Antifade Mounting Medium with DAPI (H-1200-10, Vector Laboratories, Burlingame, CA). Slides were imaged using a Zeiss 710 confocal microscope. Phagocytosis was identified using Fiji by Image J, by determining the number of GFP + macrophages containing red dye in their vesicles. Live imaging was performed following a similar protocol and images were acquired through ImageXpress Pico Automated Cell Imaging System (Molecular Devices, San Jose, CA, USA).

**Immunofluorescence staining** A31A7 cells or BMDMs derived from wild-type C57BL/6 mice were plated in Nunc Lab-Tek™ 8-well chamber slides (177402PK, ThermoFisher). The same treatment and polarization protocols as previously described were followed for these experiments. Twenty four hours after polarization, slides were fixed using 4% paraformaldehyde. The antibody against ARG-1 required an antigen retrieval step, where slides were boiled in citrate-based antigen unmasking buffer (H-3300-250, Vector Laboratories) for 10 min in a microwave. Slides were then incubated overnight at 4 °C with their respective antibodies: anti-iNOS (PA3-030 A, ThermoFisher), anti-ARG1 (93668, Cell Signaling Technologies), anti-SIRPα (13379, Cell Signaling Technologies), and anti-CD68 (137001, BioLegend). The next day, slides were incubated with goat anti-rabbit IgG conjugated to Alexa Fluor 488 (A11008, ThermoFisher) or goat anti-rat IgG conjugated to Alexa Fluor 555 (A-21434, ThermoFisher) for 1 h at room temperature. Slides were mounted using VECTASHIELD Antifade Mounting Medium with DAPI (H-1200-10, Vector Laboratories, Burlingame, CA). Slides were imaged using a Zeiss LSM 710 confocal microscope and analyzed using Adobe Photoshop.

**Flow cytometry** Immunophenotyping of tumors by flow cytometry was performed following the protocol previously described [21, 22]. When control tumors reached the endpoint (2000 mm^3^), mice were euthanized following the IACUC protocol at The George Washington University, and tumors were processed into a single-cell suspension for analysis by flow cytometry with tumor digestion buffer containing collagenases I and IV, hyaluronidase V, and DNAse I. Dead cells were discriminated using LIVE/DEAD™ Fixable Aqua Dead Cell Stain Kit (ThermoFisher Scientific, L34965). The antibodies used to stain cell surface markers were all purchased from BioLegend (San Diego, CA) unless otherwise mentioned. Myeloid cell surface markers include: APC/Fire 750 anti-mouse CD45.2 (clone 104), Brilliant Violet 421 anti-mouse/human CD11b (clone M1/70), Brilliant Violet 605 anti-mouse Ly-6G/Ly-6 C (Gr-1) (clone RB6-8C5), Brilliant Violet 785 anti-mouse F4/80 (clone BM8), APC anti-mouse CD80 (clone 16-10A1), PerCP/Cyanine5.5 anti-mouse H-2Kd (clone SF1-1.1), PE/Cy7 anti-mouse CD206 (MMR) (clone C068C2), PE anti-mouse CD163 (clone 5B11), and Alexa Fluor 700 anti-mouse CD3 (clone 17A2). T cell surface markers are as follows: Brilliant Violet 785 anti-mouse CD45.2 (clone 104), PerCP/Cy5.5 anti-mouse CD3 (clone 17A2), APC/Fire 750 anti-mouse CD8a (clone 53-6.7), Brilliant Violet 650 anti-mouse CD4 (clone GK1.5), Brilliant Violet 605 anti-mouse CD44 (clone IM7), PE anti-mouse CD62L (clone MEL-14), and Pacific Blue anti-mouse CD25 (clone PC61). NK cell surface markers are as follows: Brilliant Violet 785 anti-mouse CD45.2 (clone 104), PerCP/Cy5.5 anti-mouse CD3 (clone 17A2), PE/Cy7 anti-mouse CD49b (pan-NK cells) (clone DX5), Brilliant Violet 421 anti-mouse CD16/32 (clone 93), Alexa Fluor 700 anti-mouse CD8a (clone 53-6.7), and PE anti-mouse 4-1BB (clone 17B5). Multicolor flow data was acquired using a BD FACS Celesta Cell Analyzer, and data analysis was performed with FlowJo software version 10.3. The gating strategies for each immune cell panel can be found in Supp. Figure 7.

Cell surface expression of CD47 in melanoma cells was evaluated using PE anti-mouse CD47 (clone miap301, 127508200, BioLegend), PE anti-human CD47 (clone CC2C6, 323108, BioLegend), and their respective isotype controls (400508, 400113, respectively, BioLegend). SIRPα was evaluated using APC anti-mouse CD172a (SIRPα) antibody (clone P84, 144014, BioLegend) or APC anti-human CD172a/b (SIRPα/β) antibody (clone SE5A5, 323810, BioLegend) and their respective isotype controls (400411, BioLegend).

### Titration of αCD47 in vitro

The blockade and saturation of the mouse anti-CD47 blocking antibody were evaluated following the protocol described by Yang Li et al. [46]. Briefly, anti-mouse CD47 (clone miap301, 127502, BioLegend) was serially diluted to block cell surface CD47 in SM1 melanoma cells. After incubation for 1 h, cells were washed and stained with PE anti-mouse CD47 (clone miap301, 127508200, BioLegend) to evaluate free cell surface CD47 or with PE anti-rat IgG2a antibody (clone Poly4054, 405406, BioLegend) to evaluate CD47 antigen saturation after blockade.

**qRT-PCR** Total RNA was extracted from cells using the Trizol reagent according to manufacturer’s instructions (ThermoFisher Scientific, 15596018). Samples were then processed immediately or stored at − 80 °C. RNA was quantified using a NanoDrop One Spectrophotometer (ThermoFisher Scientific). The 260/280 ratios were routinely over 1.8. Sample cDNA was produced using the iScript cDNA synthesis kit (Bio-Rad, 1708891). Target mRNA was quantified using MyIQ single-color real-time PCR detection system from Bio-Rad (Bio-Rad, Hercules, CA) and iQ SYBR green Supermix (Bio-Rad, 1708882). The following primers were synthesized by Invitrogen (Waltham, MA) with sequences designed by Origene (Rockville, MD): mouse β-actin (MP200232), mouse Nos2 (MP208933), mouse MRC1 (MP207800), human β-actin (HP204660), human IL-1β (HP200544), human MRC1 (HP206121), human NOS2 (HP200591), human CD209 (HP214086), mouse SIRPα (MP215450), mouse LRP1 (MP207604), human LRP1 (HP206040), mouse MFGE8 (MP208190), human MFGE8 (HP225757), mouse CD36 (MP201923), human CD36 (HP200058), and mouse CD47 (MP201932). Primers for mouse ARG-1 (PPM31770C) and human SIRPα (QT01031051) were purchased from Qiagen (Hilden, Germany). Human CD47 primer sequences were obtained from Sudo T, *et al* and are 5′-GGCAATGACGAAGGAGGTTA-3′ (sense) and 5′-ATCCGGTGGTATGGATGAGA-3′ (antisense) [62]. Human CD86 primer sequences are 5’-CAACACAATGGAGAGGGAAGA-3’ (sense) and 5’-TTAAAAACACGCTGGGCTTC-3’ (antisense). Mouse CD80 sequences are 5’-GATGCTCACGTGTCAGAGGA-3’ (sense) and 5’-CAACGATGACGACGACTGTT-3’ (antisense). Cycling conditions were set as per the manufacturer’s instructions. Single product amplification was confirmed by melting curve analysis. Quantification is expressed in arbitrary units and target mRNA levels were normalized to β-actin expression.

**Immunoblotting** Cells were treated with the respective compounds for 24 h. Then, cells were lysed using RIPA buffer (ThermoFisher Scientific Pierce, 89,901, Waltham, MA) supplemented with 1X protease and phosphatase inhibitor (ThermoFisher Scientific, A32961). Lysates were sonicated at 4 °C for 8 min (8 cycles of 30s on, 30s off). Protein concentration was quantified using a BCA protein assay (ThermoFisher Scientific, 23225) following manufacturer’s instructions. Samples were mixed with 6X Laemmli SDS sample reducing buffer (Alfa Aesar, J61337AC, Tewksbury, MA), and boiled at 80 °C for 10 min. Next, samples were loaded onto 4–20% gels (Bio-Rad, 4561096, Hercules, CA), transferred to PVDF membranes and blocked with Odyssey Blocking Buffer (LI-COR Biosciences, 927-40000, Lincoln, NE). The following primary antibodies used for immunoblotting were purchased from Cell Signaling Technologies (Danvers, MA): tubulin (3873), acetylated tubulin (3971), arginase-1 (93668), and SIRPα (13379). The primary antibody recognizing HDAC6 was purchased from Assay Biotechnology (C0226, Fremont, CA), and iNOS antibody was purchased from ThermoFisher Scientific (PA3-030 A). Bands were detected using Azure Biosystems Imaging System c600 (Azure Biosystems, Dublin, CA).

**Gene expression analysis** Correlations between CD47 or SIRPα expression and HDAC6 expression or overall survival of skin cutaneous melanoma patients were obtained through Gene Expression Profiling Interactive Analysis (GEPIA, http://gepia.cancer-pku.cn/) [35] Overall survival was determined using a quartile cutoff for expression (75% high vs. 25% low), and correlation between HDAC6 and CD47 or SIRPα expression was determined by Pearson correlation.

### Luciferase assays

The mouse CD47 or SIRPα promoters were cloned into a promoterless pGL4.20[*luc2*/puro] vector using Genentech’s cloning services (San Francisco, CA, USA). Vectors were transfected into SM1 and B16 melanoma cells using Lipofectamine 3000 (L3000008, ThermoFisher) and transfected cells were selected using puromycin. A31A7 macrophages were transiently transfected using jetOPTIMUS DNA transfection reagent (Polyplus, NY, USA). Luminescence was measured using the Luciferase Assay System (E4550, Promega) in a SpectraMax i3x Multi-Mode Microplate Reader (Molecular Devices).

### Statistical analysis

Data were analyzed using GraphPad Prism (version 7; San Diego, CA) and presented as mean values ± SD or mean values ± SEM from a minimum of three experimental replicates. Representative data is presented from experiments that were performed at least two or three times. Tumor growing curves were analyzed by two-way ANOVA among groups. The level of significant differences in group means was assessed by student’s t-test or by two-way ANOVA, and a p value of ≤ 0.05 was considered significant in all analyses herein.

### Electronic supplementary material

Below is the link to the electronic supplementary material.

Supplementary Material 1

## Data Availability

The datasets used and/or analyzed during the current study are available from the corresponding author on reasonable request.

## Declarations

### Patient and public involvement

Patients or the public were not involved in the design, conduct, reporting, or dissemination plans of our research.
