# Supplementary material for: Targeting HDAC6 improves anti-CD47 immunotherapy
Source: J Exp Clin Cancer Res. 2024 Feb 27;43:60. doi: 10.1186/s13046-024-02982-4 (PMC10898070; doi:10.1186/s13046-024-02982-4)
Supplement: Supplementary file 1 — Supplementary Material 1 [file 13046_2024_2982_MOESM1_ESM.docx]

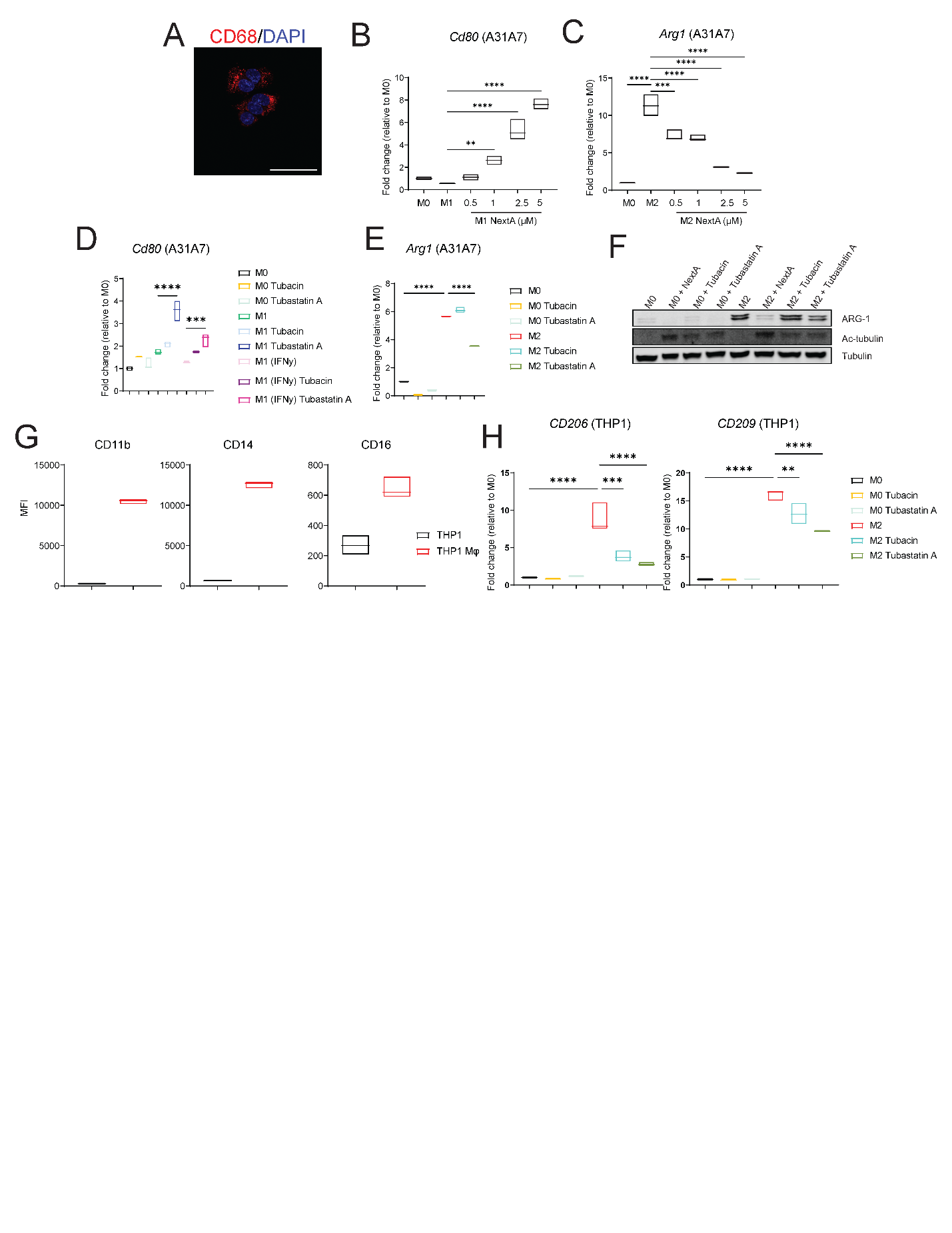


**Supp. Fig. 1. Validation of macrophage phenotype results using other HDAC6 inhibitors. A**, Immunofluorescence analysis of A31A7 macrophages stained for CD68, a pan-macrophage marker, to corroborate their macrophage identity. Nuclei stained with DAPI are shown in blue, CD68 protein staining is shown in red. **B-C**, Gene expression analysis of M1 marker *Cd80* and M2 marker *Arg1* by qRT-PCR to assess concentration-dependent effects of Nexturastat A (NextA) in modulating macrophage phenotype. **D-E**, Gene expression analysis of M1 marker *Cd80* and M2 marker *Arg1* by qRT-PCR to assess the effects of Tubacin and Tubastatin A in A31A7 macrophage polarization. **F**, Western blot analysis of A31A7 macrophages polarized to M2 to compare NextA, Tubacin, and Tubastatin A. **G**, Cell surface expression of CD11b, CD14, and CD16 by flow cytometry in THP-1 monocytes and THP-1-derived macrophages after PMA treatment. **H**, Gene expression analysis of M2 markers, *MRC1* (*CD206*) and *CD209* by qRT-PCR to evaluate the effects of Tubacin and Tubastatin A in THP-1-derived M2-like macrophage polarization. *, P<0.05; **, P<0.01; ***, P<0.001; ****, P<0.0001; ns, non-significant.


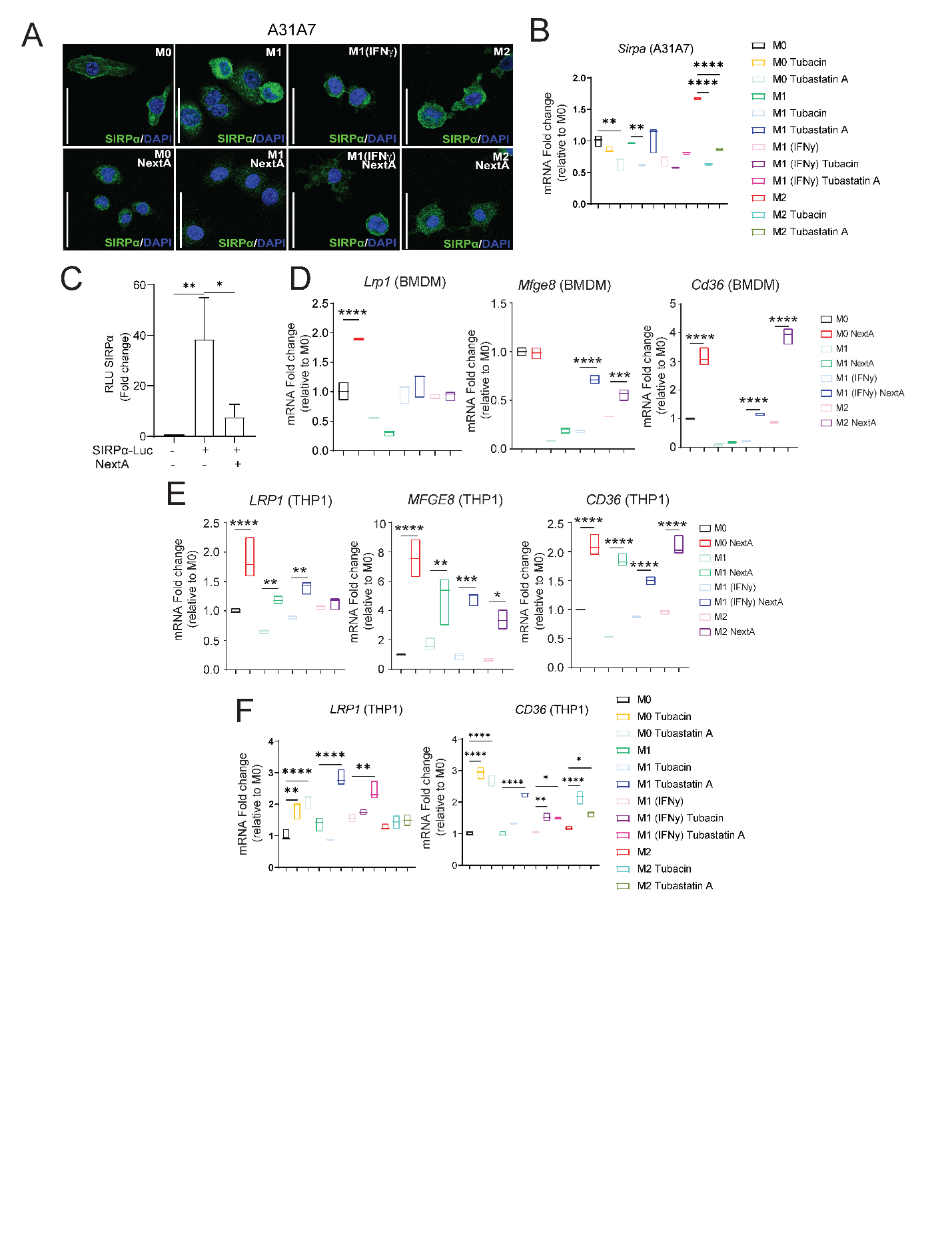


**Supp. Fig. 2. HDAC6 inhibitors regulate expression of anti- and pro-phagocytic signals on macrophages.** Macrophages were unpolarized (M0, naïve) or polarized to M1-like phenotypes and M2-like phenotype in the presence or absence of the HDAC6 inhibitors Nexturastat A (NextA), Tubacin, or Tubastatin A (TubA). **A**, Immunofluorescence microscopy representing changes in SIRPα expression (in green) in A31A7 macrophages upon NextA treatment. Nuclei were stained with DAPI (in blue). Scale bars represent 50 μm. **B**, Gene expression analysis of *Sirpα* expression in A31A7 macrophages treated with Tubacin or TubA, evaluated by qRT-PCR. **C**, Luminescence intensity measured as relative luminescence units (RLU) in A31A7 cells that were transiently transfected with SIRPα-luciferase reporter plasmid (SIRPα-luciferase) in the absence or presence of NextA. **D**, qRT-PCR of pro-phagocytic genes *Lrp1, Mfge8,* and *Cd36* in BMDMs that were untreated or treated with NextA. **E**, qRT-PCR of pro-phagocytic genes *LRP1, MFGE8,* and *CD36* in THP-1-derived macrophages that were untreated or treated with NextA. **E**, qRT-PCR of pro-phagocytic genes *LRP1, MFGE8,* and *CD36* in THP-1-derived macrophages that were untreated or treated with Tubacin or TubA. *, P<0.05; **, P<0.01; ***, P<0.001; ****, P<0.0001; ns, non-significant.

**
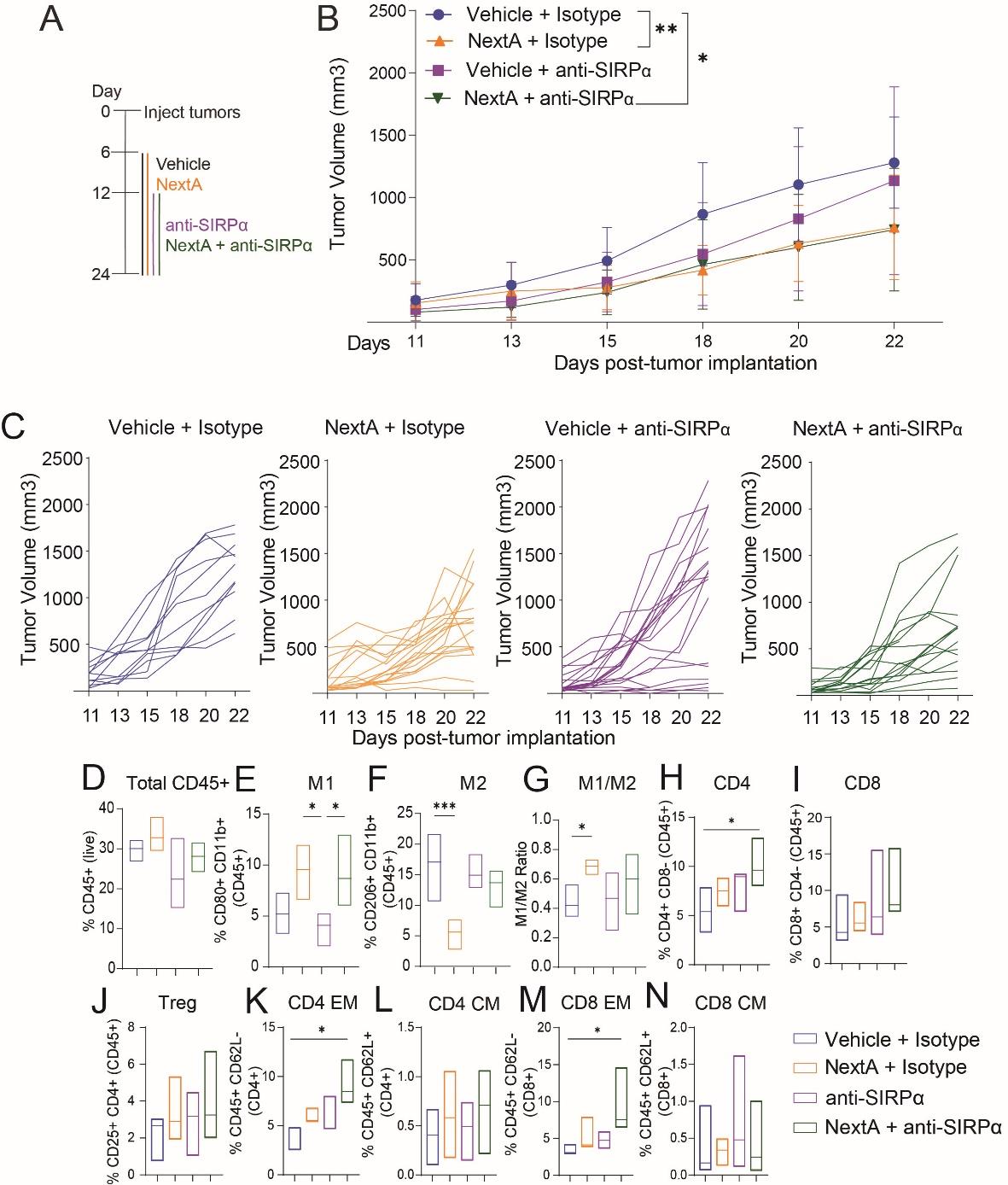
**

**Supp. Fig. 3. Combination of Nexturastat A and anti-SIRPα in SM1 melanoma-bearing mice**. **A**, Schematic representation of the therapeutic timeline. **B**, Tumor growth kinetics of SM1 melanoma tumors (n=11-19 mice per group) treated with vehicle and isotype controls, Nexturastat A (NextA, 20 mg/kg, intraperitoneal) and isotype control, anti-SIRPα (100 μg intratumoral), or combination of NextA and anti- SIRPα. **C**, Individual tumor growth kinetics in the different treatment groups. Immunophenotyping of tumors was performed by flow cytometry 22 days post-tumor implantation. The immune cell populations include total CD45+ immune cells (**D**); M1-like, M2-like, and M1/M2 ratio (**E-G**), total CD4+ T cells (**H**), total CD8+ T cells (**I**), T-regs (**J**), effector memory (EM) and central memory (CM) CD4+ T cells (**K-L**), and effector memory (EM) and central memory (CM) CD8+ T cells (**M-N**). The surface markers used to identify the populations are indicated on the y axis of every graph. The data presented in this figure represent two independent studies. *, P<0.05; **, P<0.01; ***, P<0.001; ****, P<0.0001; ns, non-significant.


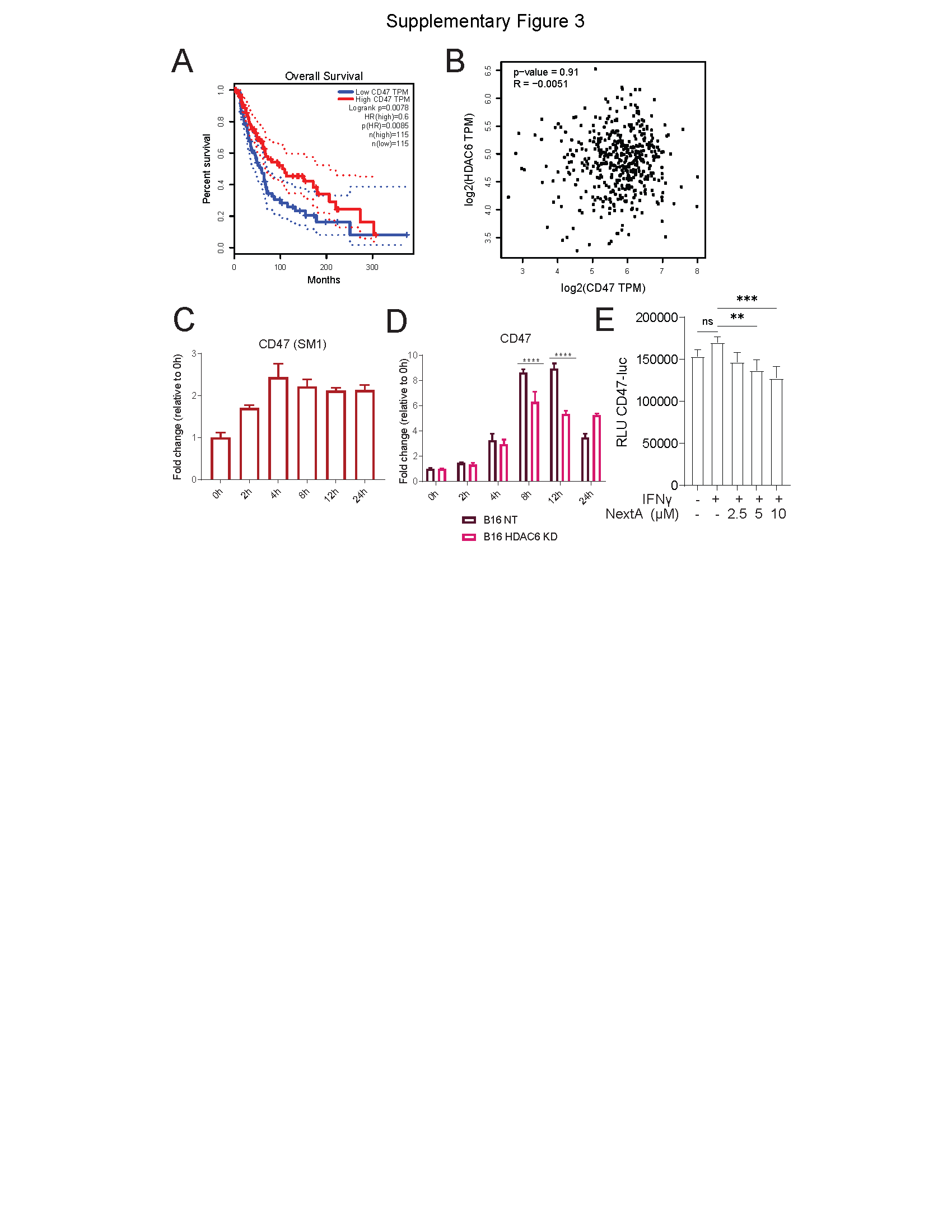


**Supp. Fig. 4. CD47 gene expression analysis in melanoma. A**, Overall survival of skin cutaneous melanoma patients as it relates to high or low CD47 expression. Data was obtained from the TCGA database through GEPIA. **B**, Pearson correlation between HDAC6 and CD47 expression in skin cutaneous melanoma patients. Data was obtained from the TCGA database through GEPIA. **C**, Gene expression kinetics evaluating *Cd47* expression in SM1 melanoma cells upon IFNγ stimulation, as evaluated by qRT-PCR. **D**, Gene expression kinetics evaluating *Cd47* expression in B16 melanoma cells non-target control (NT) or HDAC6 knockdown (KD) upon IFNγ stimulation, as evaluated by qRT-PCR. **E**, Luminescence intensity measured as relative luminescence units (RLU) of SM1 cells transiently transfected with CD47-luciferase (CD47-luc) reporter plasmid upon IFNγ stimulation in the absence or presence of NextA at different concentrations. *, P<0.05; **, P<0.01; ***, P<0.001; ****, P<0.0001; ns, non-significant.


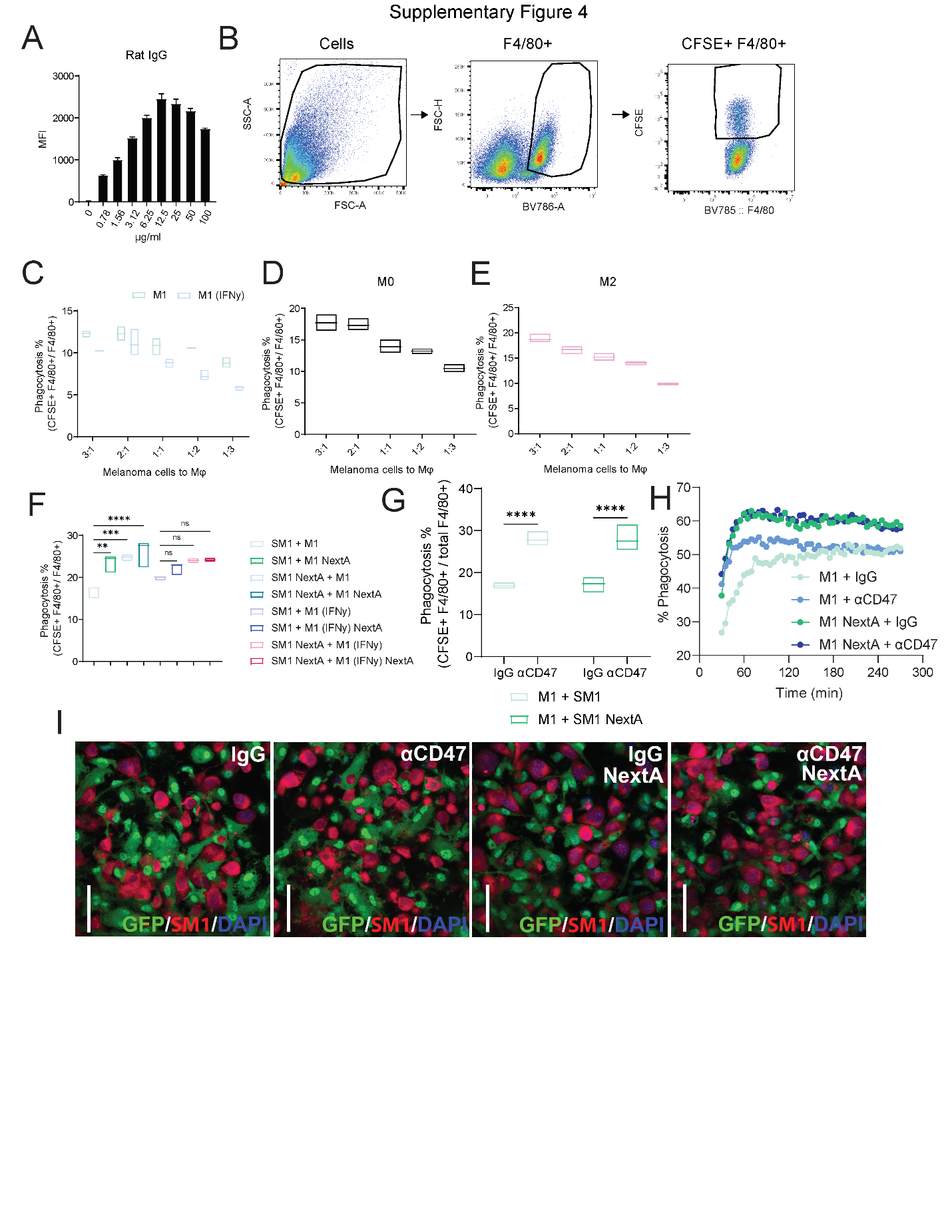


**Supp. Fig. 5. Nexturastat A improves phagocytosis of melanoma cells and prevents M1 macrophage phenotype switch towards M2. A**, CD47 epitope saturation upon treatment of SM1 melanoma cells with increasing concentrations of anti-CD47 (miap301), evaluated by flow cytometry. **B**, Representative gating strategy for flow cytometry-based phagocytosis assays. Figure panels C through G represent phagocytosis assays performed by flow cytometry. **C-E**, Phagocytosis assays at different ratios of B16 melanoma cells to A31A7 macrophages( 3:1, 2:1, 1:1, 1:2, 1:3) that were unpolarized (M0) or polarized to M1-like or M2-like phenotypes to determine the best ratio for phagocytosis assays. **F**, Phagocytosis assay of SM1 cells and M1-like A31A7 macrophages in the presence or absence of NextA. **G**, Phagocytosis assay of SM1 untreated or NextA cells that were cocultured with M1-like BMDMs in the presence of IgG control or anti-CD47. This was tested in parallel to the data presented in Figure 4E. **H**, Kinetics of phagocytosis of M1-like BMDMs, untreated or NextA treated, were cocultured with SM1 cells in the presence of isotype control or anti-CD47, as evaluated by live cell imaging for 5 hours. **I**, Unmarked version of the confocal images shown in Fig. 5K. Scale bars represent 50 μm. *, P<0.05; **, P<0.01; ***, P<0.001; ****, P<0.0001; ns, non-significant.


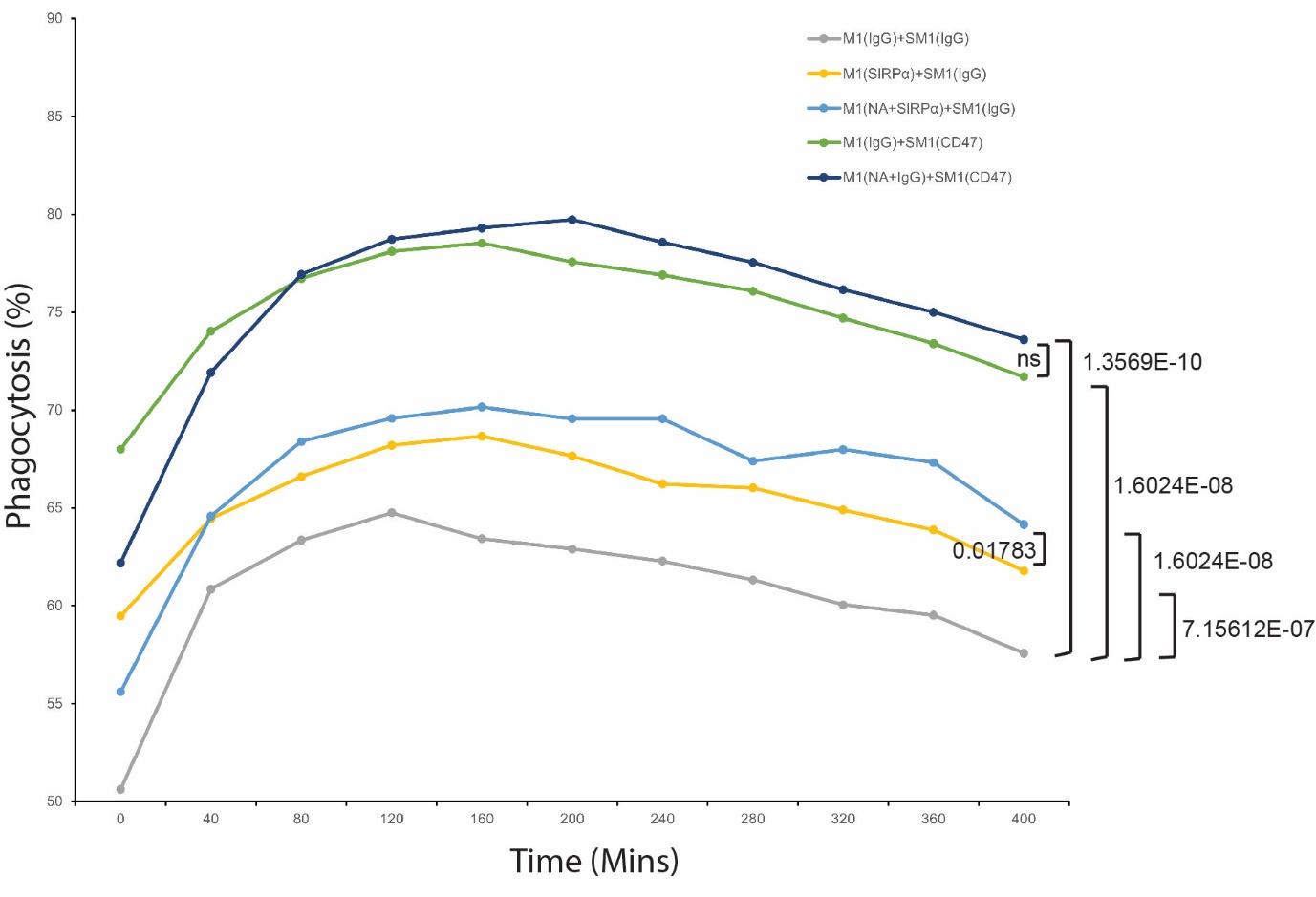


**Supp. Fig. 6. Phagocytosis of SM1 melanoma cells by bone marrow derived macrophages.** Kinetics of phagocytosis evaluated by live cell imaging. GFP expressing murine BMDMs were polarized to M1 macrophages and were left untreated or were treated with Nexturastat A (NA). Macrophages were cocultured with SM1 melanoma cells in the presence or absence of SIRPα or CD47 blocking antibodies, or their respective isotype controls (IgG). SM1 melanoma cells were stained with CellTrace Far Red. Blocking CD47 on SM1 cells significantly increased phagocytosis by M1 macrophages (dark blue and green lines) compared to blocking SIRPα (cyan and yellow lines).

**
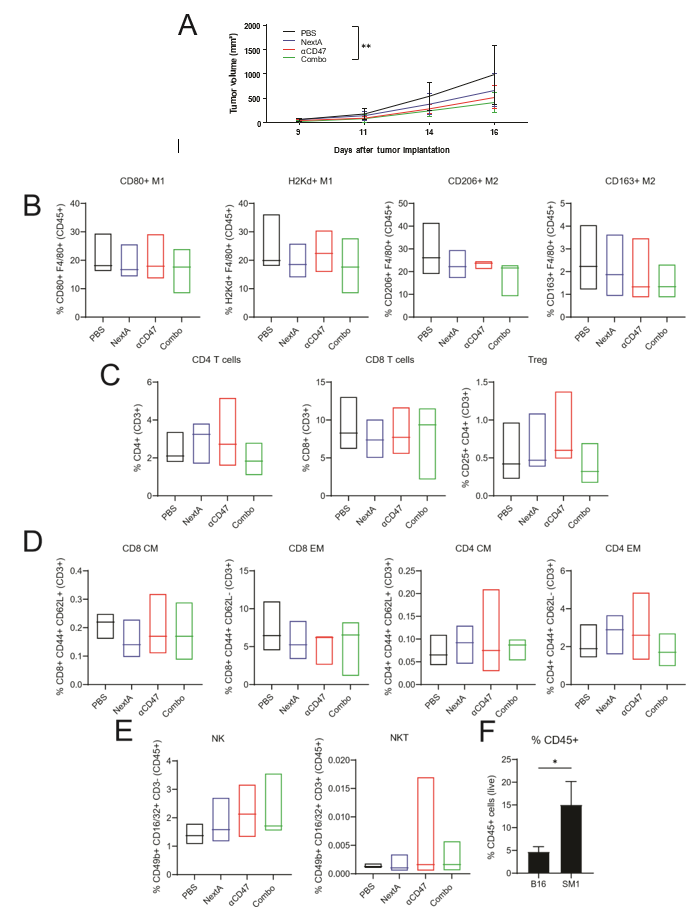
**

**Supp. Fig. 7. Combination of Nexturastat A and anti-CD47 in B16-bearing mice. A**, Tumor growth of B16F10 melanoma tumors (n=15 mice per group) treated with vehicle control, Nexturastat A (NextA, 25 mg/kg, IP), αCD47 (50 μg IT), or combination. Immunophenotyping of tumors was performed by flow cytometry 20 days post-tumor implantation. Different M1-like and M2-like macrophage populations (**B**), T cell populations (**C, D**) and NK cell (**E**) populations were identified. The respective surface markers used to identify these immune populations are indicated on the Y-axis of each graph. **F**, Comparison of total CD45+ immune cell infiltration in B16 and SM1 tumors. *, P<0.05; **, P<0.01; ***, P<0.001; ****, P<0.0001; ns, non-significant.

**
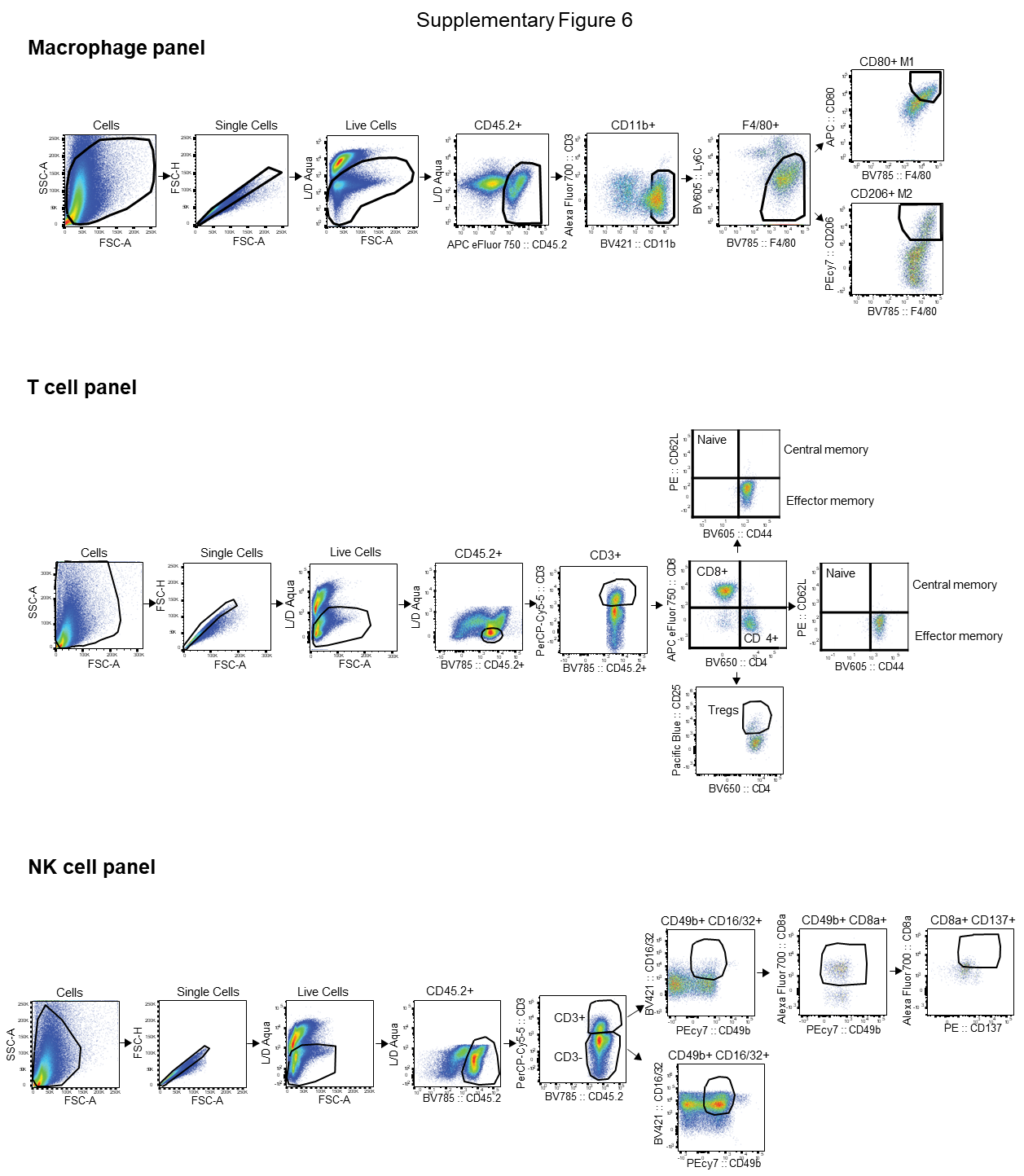
**

**Supp. Fig. 8. Gating strategies for flow cytometry-based immunophenotyping of tumors.** Gating strategies utilized to identify multiple macrophage, T cell, and NK cell populations in tumors harvested from mice in the combination therapy studies.
